# A modern and practical laccase-catalysed route suitable for the synthesis of 2-arylbenzimidazoles and 2-arylbenzothiazoles

**DOI:** 10.1039/c8ra07377e

**Published:** 2018-11-27

**Authors:** Mudzuli Maphupha, Wanyama P. Juma, Charles B. de Koning, Dean Brady

**Affiliations:** Molecular Sciences Institute, School of Chemistry, University of the Witwatersrand Johannesburg South Africa dean.brady@wits.ac.za +27-11-7176745

## Abstract

Heterocyclic aromatic compounds containing an imine (C=N) bond such as benzimidazoles and benzothiazoles are important active pharmaceutical ingredients. The synthesis of 2-aryl-1*H*-benzimidazoles and 2-arylbenzothiazoles in good to excellent yields was achieved by reacting 2-aminoaromatics with various benzaldehyde derivatives catalysed by the commercial laccases Novoprime and Suberase® at room temperature and in the presence of atmospheric oxygen.

## Abstract 2

Green chemistry: laccase in acetonitrile and buffer in the presence of O_2_ can synthesise benzimidazoles and benzothiazoles in good yields.

## Graphical abstract

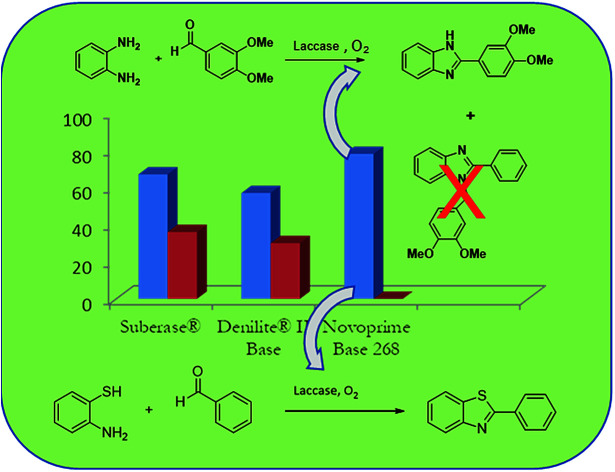

## Introduction

Applying enzymes to address modern challenges experienced in complex synthetic organic chemistry has proven valuable. Enzymes provide alternative and sustainable processes and have helped to minimize the release of hazardous substances into the environment.^1^ Laccases (benzenediol : oxygen oxidoreductases, EC1.10.3.2) are well recognized oxidoreductase enzymes belonging to the family of blue multi-copper-containing oxidases.^1^ They are capable of catalysing oxidation reactions of several low molecular weight organic compounds such as polyphenols, aminophenols, methoxyphenols, aminophenols, and lignin-related molecules.^1–4^ The catalytic process of these enzymes occurs through a one-electron oxidation and water is released as the by-product.^1^ Because of their broad catalytic activity, laccases have been utilised in a variety of industrial applications ranging from textile to the pulp and paper industries and from food industries to bioremediation processes.^5^

**Fig. 1:**
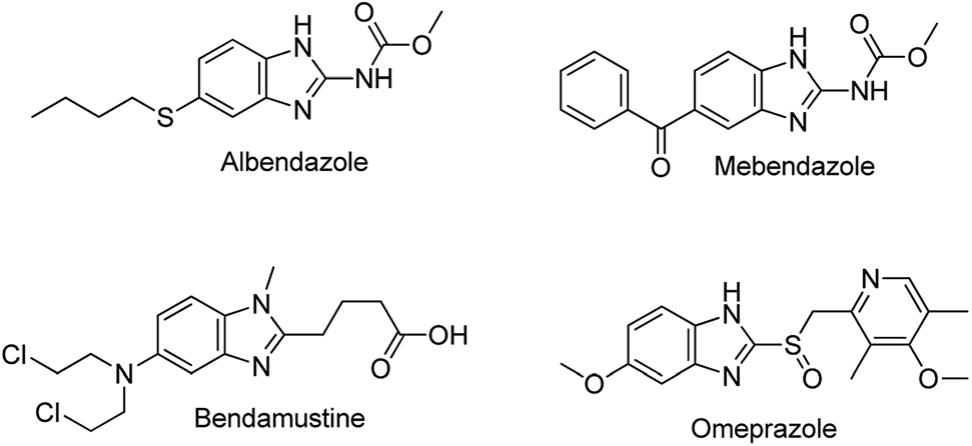
Pharmaceuticals a benzimidazole nucleus.

Benzimidazoles are a class of heterocyclic aromatic compounds composed of an imidazole ring fused to a benzene ring. Because of their remarkable biological activities, benzimidazoles have been studied for more than a century and are a common moiety in many active pharmaceuticals (Fig. 1). One of the prominent natural occurring derivatives of benzimidazoles is *N*-ribosyl-dimethyl benzimidazole; a component of inhibitors such as factor Xa (Fxa) inhibitors, poly (ADP-ribose) polymerase (PARP) inhibitors, 5-HT3 antagonists, and 5-lipoxygenase inhibitors, and also serves as one of the cobalt ligands in vitamin B12.^6^ Substituted benzimidazoles are associated with a wide range of biological and pharmacological activities such as anti-tumour, anti-ulcer, anti-fungal, anti-hypersensitive, antiviral agents, anti-allergic properties, as well as neuropeptide Y Y1 receptor antagonists.^7,8^ Furthermore, through blocking the secretion of gastric acid in the stomach, substituted benzimidazoles such as omeprazole can act as gastric H^+^/K^+^ ATPase inhibitors.^9–11^ Some of the well-known available drugs bearing a benzimidazole scaffold include albendazole, mebendazole, omeprazole, and bendamustine (Fig. 1).^12^ Furthermore, compounds with benzimidazole moieties have been utilized in material science applications; they are often used as membranes for fuel cells and in organic light-emitting diodes.^13^

**Fig. 2:**
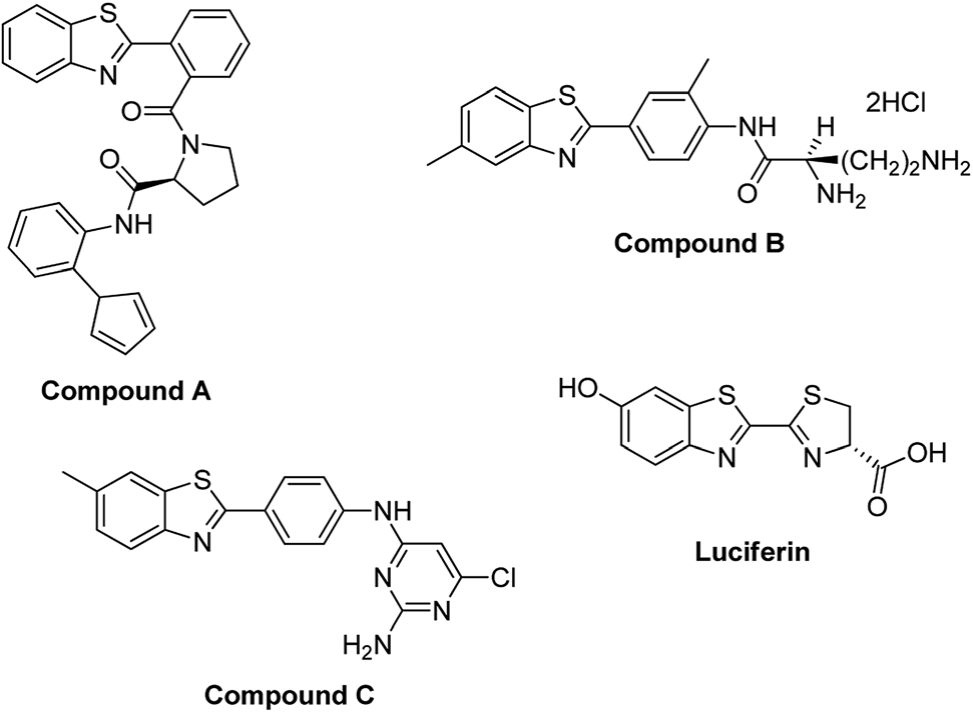
Benzothiazoles in biologically active compounds.

A related class of compounds, the benzothiazoles, are also an important class of aromatic heterocyclic compounds composed of a benzene ring fused to a thiazole ring where a thiazole ring is a five membered ring made up of sulphur and nitrogen atoms.^14,15^ The benzothiazole moiety substituted with various functional groups has attracted attention due to their pharmacological and therapeutic activities (Fig. 2).^14^ These compounds have displayed antitumor,^16^ anticancer,^14,15,17^ anti-HIV,^18^ antiviral,^19^ antimicrobial,^20^ antibacterial,^21^ anthelmintic,^22^ anti-diabetic,^23^ anti-allergic^24^ and anti-inflammatory activities.^25^ Other reported applications include use in industry as oxidants, industrial dyes and functional materials.^16,26–33^

Due to their numerous biological activities, development of efficient synthesis methods for benzimidazoles and benzothiazoles compounds has received a significant amount of interest.^7,12,14^ The conventional methods include condensation–dehydration reaction of *ortho*-aminoaromatics with carboxylic acids and their derivatives under strong acid and high temperature conditions or condensation with aldehydes under oxidative conditions.^26,34^ A number of oxidants have been used to catalyse these reactions, including iodine, peroxides, 1,4-benzoquinone, nitrobenzene, cupric acetate, and (bromodimethyl)sulfonium bromide, and recent advances for benzimidazoles include procedures such as microwave assisted synthesis and solid phase synthesis.^10^ The use of transition-metal catalysed cross-couplings reactions of benzothiazoles and aromatic halides using aromatic boronic and carboxylic acids is also known.^27^

Most reported methods are disadvantageous because they involve use of toxic oxidants, abrasive reaction conditions (high temperatures and pressure, as well as long reaction times). Furthermore, the transition metals are expensive while solvents such as NMP, DCM, and DMF are environmentally unfriendly.^26,27^

Greener approaches have been reported recently; this includes the laccase catalysed reaction between *o*-phenylenediamine and benzaldehydes as well as the two-step laccase-mediator aerobic oxidation condensation of *in situ*-produced salicylaldehyde derivatives with aromatic amines for the synthesis of benzimidazole derivatives.^7^

Therefore, alternative and more environmental friendly preparation of 2-aryl-1*H*-benzimidazoles and 2-aryl-benzothiazoles involves enzymes.^1,7^ Herein, we report laccase catalysed one-pot syntheses of 2-aryl-1*H*-benzimidazoles and 2-arylbenzothiazoles from a condensation–dehydration reaction of 2-aminoaromatics with aryl-aldehydes.

## Results and discussion

**Scheme 1:**
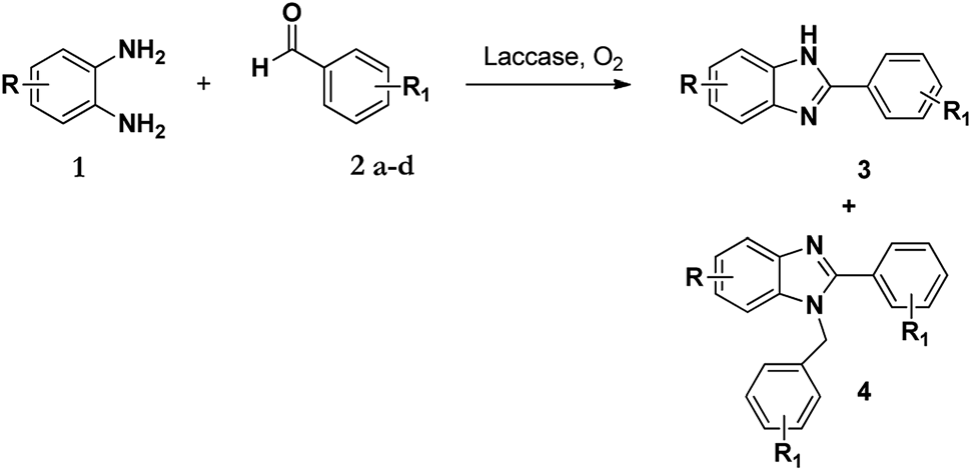
Laccase catalysed reaction of 1 and 2 in acetate buffer (pH 4.5) using various co-solvents.

The synthesis of benzimidazole derivatives can be achieved through reactions of derivatives of *o*-phenylenediamine 1 with aldehydes 2 with using molecular oxygen as an oxidant. However, this results not only in the formation of 2-substituted benzimidazole (3) but also 2,3-disubstituted benzimidazole (4) as a by-product or even the major product (Scheme 1).^7^ Hence, although this protocol was effective, poor chemoselectivity was a disadvantage. Recently, Leutbecher *et al.* discovered that the same reaction using the laccase catalysed enzyme domino reaction between *o*-phenylenediamine 1 with various aromatic aldehydes 2 in aerobic conditions using a phosphate buffer and obtained 2-aryl-1*H*-benzimidazoles 3 in good to excellent yields but with some di-substituted by-product 4.^7^

It has been shown that in the presence of an oxidizing agent the reaction of 1 with 2 favours the formation of product 3 over product 4.^34^ Following this, our aim was to first identify how and why the 1-*N*-benzylated product 4 was formed and if its formation could be controlled and possibly eliminated. Our initial approach was to investigate the selectivity of these enzyme-facilitated reactions by varying the pH, types of buffers and co-solvents used in the synthesis of these compounds. The co-solvents used in this study were methanol, ethanol, DMF, and acetonitrile and the buffers were acetate and phosphate at various pH values.

**Fig. 3:**
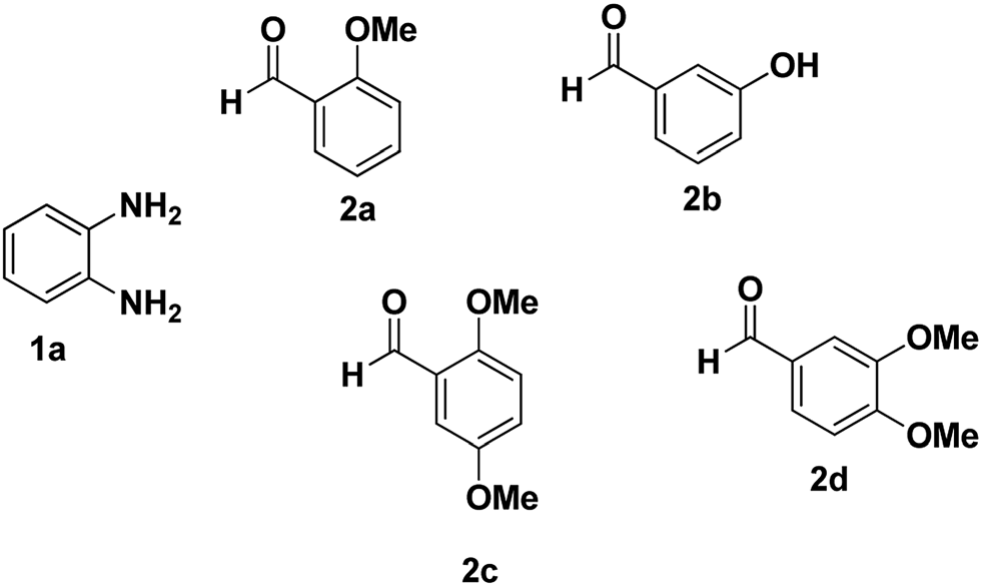
*o*-Phenylenediamine (1a) and aromatic aldehydes (2a–d) used in this study.

As a starting point, we investigated the reaction between *o*-phenylenediamine (1a) with benzaldehydes 2a–d (Fig. 3) (1 : 2 ratio) in acetate buffer (pH 4.5) using the laccase Suberase® from Novozymes at room temperature (Scheme 1). Initially, the reaction times for these reactions varied from 2 to 24 h; it was observed that for the majority of the substrates, the mono-substituted product (3) forms first and then followed by the undesired *N*-benzylated product (4). Therefore, a reaction time of 24 h was selected for the model studies to detect the formation of by-product compound 4.

Secondly, the relative ratios of products 3 and 4 obtained by laccase-catalysed oxidation were profoundly influenced by the type of organic solvent used. As has been observed previously inferior results were obtained while using methanol and ethanol as co-solvents, as they both showed a great deal of competitive formation of products 3 and 4.^7,12^ Improved chemoselectivity was observed with DMF and acetonitrile; wherein we were able to minimize the formation of the 1-*N*-benzylated product 4 (Table 1).

**Table 1:** Suberase®-catalysed formation of benzimidazoles through reaction of *o*-phenylenediamine (1a) and benzaldehydes 2a–d in acetate buffer (0.1 M, pH 4.0) using various co-solvents^a^

Entry	Aldehyde	Co-solvent	Time	3 Yield %	4 Yield %
1	2a	Methanol	24 h	59	20
2	2a	Ethanol	24 h	—	13
3	2a	DMF	24 h	72	—
4	2a	Acetonitrile	24 h	62	—
5	2b	Methanol	24 h	—	—
6	2b	Ethanol	24 h	—	97
7	2b	DMF	24 h	—	98
8	2b	Acetonitrile	24 h	—	82
9	2c	Methanol	24 h	10	90
10	2c	Ethanol	24 h	25	70
11	2c	DMF	24 h	—	74
12	2c	Acetonitrile	24 h	—	56
13	2d	Methanol	24 h	34	66
14	2d	Ethanol	24 h	—	—
15	2d	DMF	24 h	67	23
16	2d	Acetonitrile	24 h	59	36

a Reaction conditions: *Myceliophthora thermophilia* laccase (2.0 mL Suberase®) 50 : 50 solvent/acetate buffer at 25 °C.

Thirdly, we explored the possible effect of the type of laccase used on selectivity of these reactions. When Fan *et al.* developed a highly chemoselective scandium(ii) triflate based method for synthesis of either 2-benzimidazoles or 2,3-benzimidazoles it was demonstrated that the presence of a strong oxidizing agent such as hydrogen peroxide influences the production of the 2-substituted benzimidazoles over the 2,3-disubstituted benzimidazoles. This implies that the redox potential of the laccase used could influence the chemoselectivity of the reactions.^35,36^

Therefore, using laccases (Suberase®, Denilite® II Base, and Novoprime Base 268) from Novozymes, we investigated the role of enzyme type and preparation on these reactions. The reaction between *o*-phenylenediamine (1a) with benzaldehyde 2d was chosen as a model reaction and it was performed using an acetate buffer (pH 4.5) and acetonitrile at room temperature (Scheme 1).

**Fig. 4:**
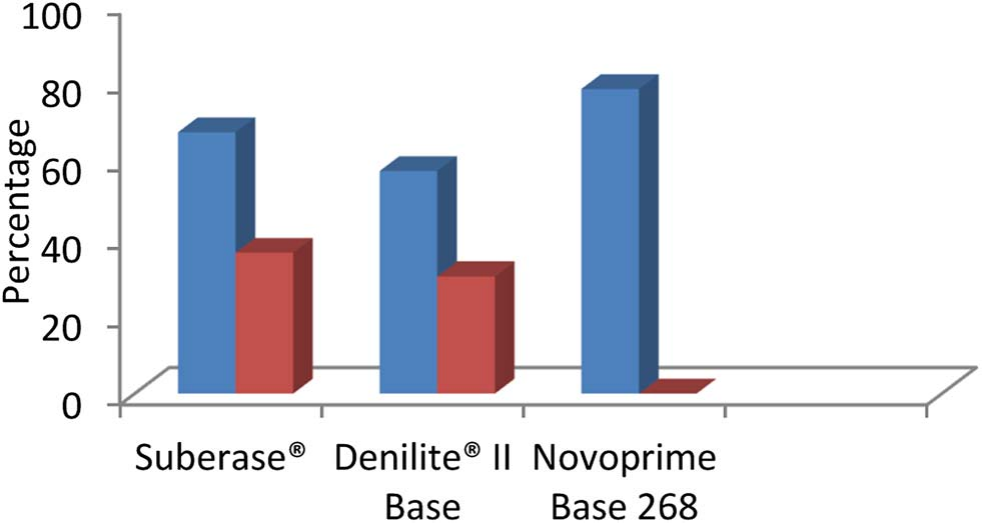
Comparison of commercial laccase preparations in the chemo-selective synthesis of 2-benzimidazole (blue) and the by-product 2,3-benzimidazole (red).

The findings shown in Fig. 4, clearly indicate that the specific laccase influences the chemoselectivity of benzimidazole derivatives to a great extent, based on the redox potential of each enzyme. Novoprime Base 268 presented the best oxidation results compared to the two laccases preparations from *Myceliophthora thermophile* (Suberase® and Denilite® II Base) for the formation of 2-(3,4-dimethoxyphenyl)-1*H*-benzimidazole (3d). The reaction conducted using Suberase® as our laccase afforded a yield of 59% of the 2-substituted benzimidazole 3d and 36% was the 3,4-disubstituted benzimidazole 4d. When using Denilite® II Base as our laccase, the ratio was 57% compound 3d and 30% compound 4d. Finally, when the enzyme Novoprime Base 268 was used, compound 3d was formed in a 78% yield while compound 4d was not detected under these reaction conditions.

Finally, in order to identify the optimal conditions for these reactions we also considered the effects of using different buffers and pH towards the reaction selectivity. Leutbecher *et al.* investigated the effect of different buffers using acetate and phosphate buffers while varying buffer to co-solvent ratios. It was shown that the ratios of buffer to co-solvent affect chemo-selectivity and consequently the yield of the products formed.^7^ In this study the buffer and pH studies were conducted using phosphate buffer (0.1 M, pH 6.0–7.5) and acetate buffer (0.1 M, pH 3.0–5.0) with DMF or acetonitrile as co-solvents at room temperature. Reactions at lower pH values (3.0–5.0) occurred at faster rates than reactions carried out at higher pH values (>7.15), but no significant effect on selectivity was observed.

Using the discovered optimal conditions (General method), derivatives of *o*-phenylenediamine were treated with a series of aromatic aldehydes using commercial laccase, Novoprime Base 268, from Novozymes at room temperature and the obtained results are summarised in Table 2 below.

**Table 2:** Novoprime-catalysed formation of benzimidazoles through reaction of *o*-phenylenediamines (1 equiv.) and benzaldehyde derivatives (1 equiv.) in acetonitrile and acetate buffer (pH 4.0) at room temperature^a^

Entry	Amine	Benzaldehyde	Time	Product	Yield %
1	R = H	R_1_ = 2-OMe	24 h	3a	69
2	R = H	R_1_ = 3-OH	24 h	4b	82
3	R = H	R_1_ = 2,5-diOMe	24 h	3c	76
4	R = H	R_1_ = 3,4-diOMe	24 h	3d	78
5	R = H	R_1_ = H	2 h	3e	92
6	R = H	R_1_ = 2-Cl	2 h	3f	94
7	R = H	R_1_ = 4-Cl	4 h	3g	88
8	R = H	R_1_ = 2-NO_2_	8 h	3h	56
9	R = H	R_1_ = 2-(Pyridin-4-yl)	2 h	3i	60
10	R = H	R_1_ = 3-NO_2_	4 h	3j	95
11	R = H	R_1_ = 4-(Dimethylamino)	8 h	3k	64
12	R = H	R_1_ = 4-NO_2_	2 h	3l	94
13	R = H	R_1_ = 3,4,5-TriOMe	24 h	3m	89
14	R = H	R_1_ = 4-OMe	24 h	3n	68
15	R = 4-Br	R_1_ = 2-(Pyridin-4-yl)	4 h	3o	94
16	R = 4-Cl	R_1_ = 3,4-diOMe	2 h	3p	93
17	R = 4-Me	R_1_ = H	8 h	3q	79
18	R = H	R_1_ = 2-Br, 5-OMe	3 h	3r	90

a Reaction conditions: laccase (0.105 g Novoprime Base 268) 50 : 50 acetonitrile/acetate buffer at room temperature.

As shown in Table 2, 2-aryl-1*H*-benzimidazoles were obtained in good to excellent yields (60–94%) from a variety of aromatic aldehydes. It is clear the method applies for a variety of aryl aldehydes containing both electron-donating and electron-withdrawing substituents around the ring. No by-product (4) was formed in any of the reactions. For compounds 3e, 5c and 5d by-product was previously observed by Leutbecher *et al.* in ratios of 20 : 3, 2 : 1 and 10 : 1 respectively in the absence of laccase.^7^

**Scheme 2:**
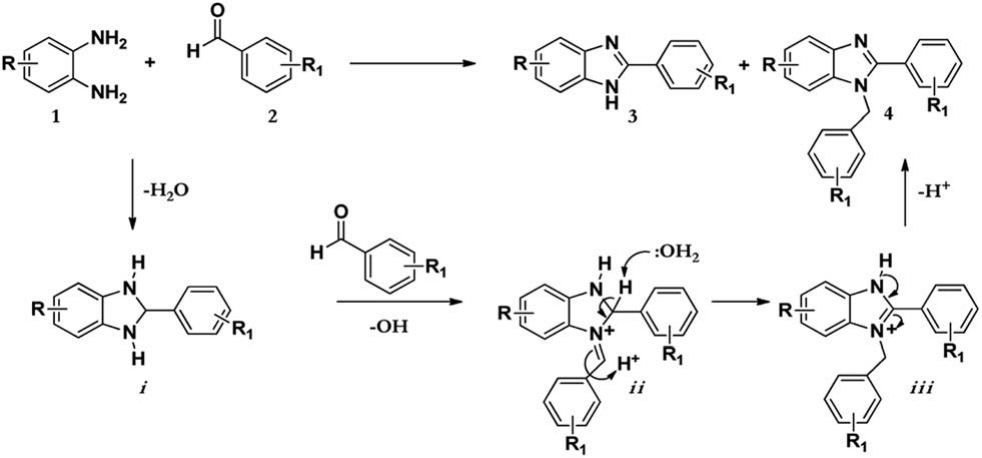
Mechanism for the synthesis of the 1,2-disubstituted benzimidazoles.

As shown in Scheme 2 below; after the condensation of the diamine and the aromatic aldehyde, the subsequent intermediate benzimidazoline i may condense further with another molecule of benzaldehyde to afford another intermediate iminium ion ii. Successively, this intermediate may tautomerize to the most stable form iii to give the resultant disubstituted benzimidazole product 4.^12^

In summary, we demonstrated that using acetonitrile in the presence of a catalytic amount of laccase (Novoprime Base 268) promotes the exclusive formation of the 2-phenyl-substituted benzimidazole for a broad range of derivatives. Our improved method is simple, selective and can afford 2-aryl-1*H*-benzimidazoles in good to excellent yields.

**Fig. 5:**
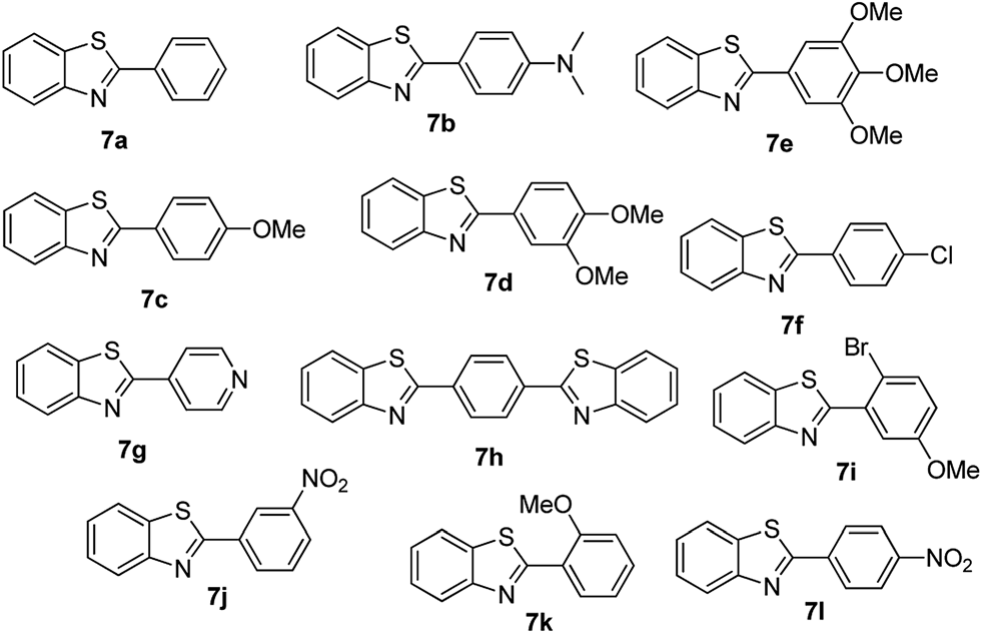
Products 7a–l of the laccase-catalysed domino reaction between 5 and 6a–l.

**Scheme 3:**
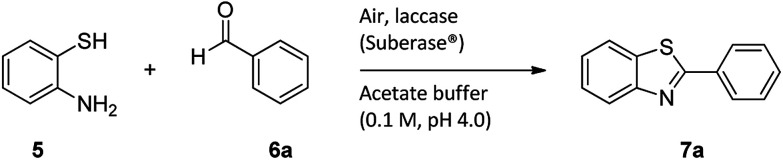
Laccase-catalysed synthesis of 2-phenyl-benzothiazole.

For the synthesis of 2-arylbenzothiazoles, we began our investigation by exploring the oxidative condensation reaction between 2-aminothiophenol (5) and benzaldehyde (6a) under aerobic conditions at room temperature. The laccase-catalysed cross-coupling reaction between 5 and 6a to afford 2-phenyl-benzothiazole (7a) (Fig. 5) in 85% yield was performed in an acetate buffer (0.1 M, pH 4.0) using acetonitrile (50%) as a co-solvent (Scheme 3 and Table 3, entry 4).

**Table 3:** Suberase®-catalysed synthesis of 2-phenyl-benzothiazole (7a) by reaction of 2-aminothiophenol (5) and benzaldehydes 6a in acetate buffer (0.1 M, pH 4.0) using various co-solvents^a^

Entry	Aldehyde	Co-solvent	Time	Product	Yield %
1	6a	Methanol	24 h	7a	58
2	6a	Ethanol	24 h	7a	55
3	6a	DMF	24 h	7a	78
4	6a	Acetonitrile	24 h	7a	85
5	6a	DCM	24 h	7a	65

a Reaction conditions: *Myceliophthora thermophilia* laccase (2.0 mL Suberase®) 50 : 50 solvent/acetate buffer at 25 °C.

The effect of organic solvents such as ethanol, acetonitrile, hexane, chloroform, DMSO and water when preparing benzothiazoles have been investigated previously.^37,38^ Sayyahi *et al.* (2015) identified ethanol as the best solvent for preparing 2-arylbenzothiazoles under reflux conditions while Gao *et al.* (2014) described water as the optimal solvent using KI catalyst.^38,39^ Therefore, in order to identify optimum reaction conditions we explored the effect of using various co-solvents. 2-Aminothiophenol (5) and benzaldehyde (6a) were reacted in the presence of a laccase (Suberase®, from Novozymes) in acetate buffer (pH 4.0) using various solvents at room temperature (Table 3).

Acetonitrile gave the best yield under the described reaction conditions. While using methanol and ethanol as co-solvents, the reaction did not go to completion after 24 h of reaction and the product (7a) (yellow oil) was difficult to isolate from the starting material (2-aminothiophenol (5)) as they both have almost indistinguishable *R*_f_ values (40% EtOAc/hexane). Conversely, Sayyahi *et al.* (2015) when using the ionic liquid [bmim][FeCl_4_] as a catalyst obtained excellent yields using ethanol but not acetonitrile.^38^

In order to purify compound 7a from the starting material, the product was dried overnight under high-vacuum to completely dry-out the oily product to an almost solid form, and cold acetonitrile was added to precipitate the product. An additional wash of cold acetonitrile (3 × 20.0 mL) was used to remove residual starting material from the product.

**Scheme 4:**
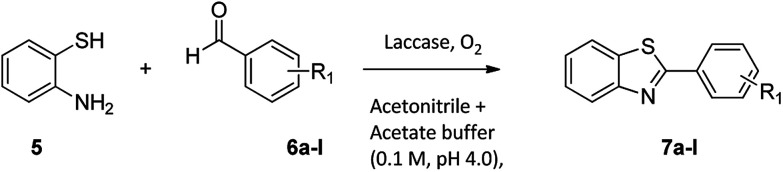
Laccase-free synthesis of benzothiazoles.

To elucidate the role of the enzyme as a catalyst, control reactions were conducted using benzaldehydes 6a–l under air in acetate buffer (0.1 M, pH 4.0)/acetonitrile (50 : 50) in the absence of laccase (Scheme 4). However only two substrates resulted in the formation of a fused product in very low yield (<10%) and no activity was observed for the remaining substrates (Table 4). Conversely, quantitative yields of the same substrates were obtained in the presence of an enzyme.

**Table 4:** Novoprime-catalysed synthesis of benzothiazoles through reaction of 1 equiv. of 2-aminothiophenol (5) with 1 equiv. of benzaldehydes 6a–1 in acetate buffer (0.1 M, pH 4.0)/acetonitrile (50 : 50) for 24 h *via*Scheme 3^a^

Entry	Benzaldehyde	Time	Product	Yield %
1	R_1_ = H	24 h	7a	85
2	R_1_ = 4-(Dimethylamino)	24 h	7b	56(8)
3	R_1_ = 4-OMe	24 h	7c	68
4	R_1_ = 3,4-diOMe	24 h	7d	73
5	R_1_ = 3,4,5-TriOMe	24 h	7e	65(7)
6	R_1_ = 4-Cl	24 h	7f	56
7	R_1_ = 2-(Pyridin-4-yl)	24 h	7g	88
8	R_1_ = 4-Acetyl	24 h	7h	87
9	R_1_ = 2-Br-5-Ome	24 h	7i	76
10	R_1_ = 3-NO_2_	24 h	7j	48
11	R_1_ = 2-OMe	24 h	7k	74
12	R_1_ = 4-NO_2_	24 h	7l	84

a Reaction conditions: laccase (0.105 g Novoprime Base 268) 50 : 50 acetonitrile/acetate buffer at room temperature.

Numbers in brackets (7b and 7e) – for these two compounds non-enzymatic limited background product formation that occurs in the absence of laccase was observed.

At this point, the most efficient method presenting the highest catalytic activity for preparing 2-arylbenzothiazoles was using acetonitrile and acetate buffer (pH 4.0), similar to the method described for 2-aryl-benzimidazole. Therefore to study the range and limitations of the optimised procedure 2-aminothiophenol was treated with a series of aryl-aldehydes in the presence of a catalytic amount of laccase to yield 2-arylbenzothiazole derivatives in good to excellent yield at room temperature (General method) and the results obtained are summarized in Table 4. Similar reactivity was observed for benzaldehydes with a variety of substituent groups; both electron withdrawing and electron donating at various positions showed comparable yields and reaction rates.

In conclusion, we have successfully developed a simple and efficient method for the synthesis of 2-arylbenzothiazole derivatives using an inexpensive commercial laccase as a catalyst at room temperature. The optimal conditions for conducting these reactions are using acetate buffer (0.1 M, pH 4.5) and acetonitrile as a co-solvent.

## Experimental

### General method used to synthesize 2-aryl-benzothiazole derivatives

A mixture of 2-aminoaromatic (10.0 mmol) and benzaldehyde derivative (10.0 mmol) in acetonitrile (10.0 mL) and acetate buffer (10.0 mL, pH 4.0) was stirred at room for 5 minutes. Laccase was then added into the mixture and the contents were stirred for 2–24 h. When the reaction completed the product precipitated from the solution and was extracted with ethyl acetate (30.0 mL) and water (3 × 20.0 mL) and concentrated on a rotary evaporator. The product was washed several times with cold acetonitrile (3 × 20.0 mL) to remove any excess starting material.

### General

All reagents and solvents were purchased from Sigma-Aldrich (South Africa) or Merck KGaA (South Africa). All solvents used for chromatographic separation were distilled before use to remove any impurities. All the chemical reagents were used as received without any further purification. Reactions were monitored by TLC carried out on Merck aluminium foil backed plates coated with silica gel (60 F254) and visualization was done under UV light. Purification of some of the compounds was done using Macherey-Nagel silica gel 60 (particle size 0.063 mm to 0.20 mm) purchased from Merck. All the melting point recordings of the compounds were performed on a Stuart SMP10 instrument. Bruker 300 and 500 MHz spectrometers were used to record both the ^1^H and ^13^C Nuclear Magnetic Resonance data using a suitable solvent at room temperature. Data processing of the spectra was done using MestreNova Software under license from Mestrelab Research, CA, USA. The resonant frequency for all spectra obtained are reported in parts per million relative to an internal standard, TMS, which appears at zero parts per million. Coupling constants are reported in Hertz. A Bruker Tensor-27 Fourier Transform spectrometer was used to perform infrared spectroscopy.

LC-MS: a 10 μL of the sample was injected into the Dionex Ultimate 3000 UHPLC system (Thermo Scientific, Dionex, Sunnyvale, California, USA) and run through a loop for one minute at 50% solvent A consisting of 0.1% formic acid in H_2_O (v/v) and 50% solvent B consisting of 0.1% formic acid in acetonitrile (v/v) at a flowrate of 0.3 mL min^−1^. High resolution mass spectra were recorded on a inked Bruker Compact Q-TOF mass spectrometer (Bruker Daltonics, Bremen, Germany) using an ESI positive source.

*Myceliophthora thermophilia* laccases (Suberase® 400 U g^−1^ and Denilite® II Base; 800 U g^−1^) and Novoprime Base 268 (origin and units not defined) were obtained from Novozymes.

### The following methods were used for the synthesis of benzimidazoles

#### Method A

A mixture of *o*-phenylenediamine (10.0 mmol, 1 equiv.) and benzaldehyde derivative (20.0 mmol, 2 equiv.) in acetonitrile (10.0 mL) and acetate buffer (10.0 mL, pH 4.5) was stirred at room temperature for 5 minutes. Suberase® (2.0 mL) was added to the mixture and the contents were stirred until reaction completes (monitored by TLC). The product precipitates from the solution as the reaction proceeds and after completion the product was extracted with ethyl acetate (30.0 mL) and water (3 × 20.0 mL) and concentrated on a rotary evaporator. The product was washed several times with cold acetonitrile (3 × 20.0 mL) to remove any excess starting material.

#### Method B

The same as Method A, except that methanol (2.0 mL) was used instead of acetonitrile.

#### Method C

The same as Method A, except that ethanol (4.0 mL) was used instead of acetonitrile.

#### Method D

The same as Method A, except that DMF (10.0 mL) was used instead of acetonitrile.

#### Method E

A repetition of Method A using Denilite® II Base (0.085 g) and Novoprime Base 268 (0.105 g) laccases instead of Suberase® (2.0 mL).

### Synthesis of 2-(2-methoxyphenyl)-1*H*-benzimidazole (3a)


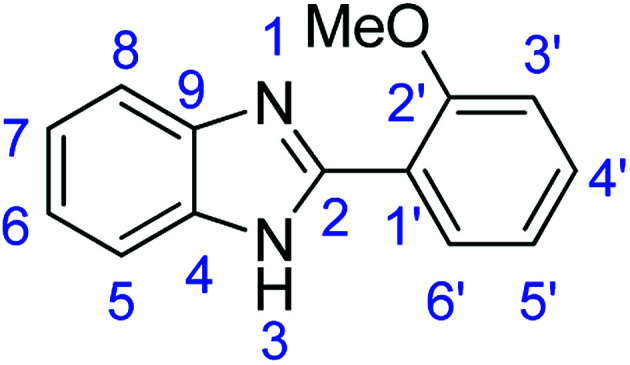


#### Method A

Stirring time = 24 h. Purification by washing with cold acetonitrile (3 × 20.0 mL) to remove any excess starting material (confirmed by TLC) to afford a brown solid (1.38 g, 62%). *R*_f_ (40% EtOAc/hexane) 0.38.

#### Method B

Stirring time = 24 h. Purification by washing with cold acetonitrile (3 × 20.0 mL) to remove any excess starting material (confirmed by TLC) to afford a brown solid (1.32 g, 59%). *R*_f_ (40% EtOAc/hexane) 0.38.

#### Method D

Stirring time = 24 h. Purification by washing with cold acetonitrile (3 × 20.0 mL) to remove any excess starting material (confirmed by TLC) to afford a brown solid (1.61 g, 72%). *R*_f_ (40% EtOAc/hexane) 0.38.

#### Method E

Novoprime Base 268 (0.105 g), stirring time = 24 h. Purification by washing with cold acetonitrile (3 × 20.0 mL) to remove any excess starting material (confirmed by TLC) to afford a brown solid (1.55 g, 69%). *R*_f_ (40% EtOAc/hexane) 0.38. Mp = 178–180 °C (lit. 40 179–180 °C). ([M + H]^+^ found: 225.1023 C_14_H_12_N_2_O requires [M + H]^+^, 225.1024). ^1^H NMR (500 MHz, DMSO-*d*_6_): *δ* 12.11 (1H, s, NH), 8.33 (1H, d, *J* = 7.6 Hz, H6′), 7.63 (2H, dd, *J* = 16.6, 7.5 Hz, H5, H8), 7.48 (1H, t, *J* = 7.8 Hz, H4′), 7.24 (1H, d, *J* = 8.3 Hz, H7), 7.19 (2H, t, *J* = 6.5 Hz, H3′, H5′), 7.12 (1H, t, *J* = 7.5 Hz, H6), 4.03 (3H, s, OMe). ^13^C NMR (126 MHz, DMSO-*d*_6_): *δ* 157.25 (C-2), 149.43 (C-2′), 143.20 (C-9), 135.19 (C-4), 131.70 (C-6′), 130.23 (C-4′), 122.51 (C-5′), 121.96 (C-1′), 121.34 (C-3′), 118.89 (C-7), 118.63 (C-6), 112.58 (C-8), 112.38 (C-5), 56.25 (OMe). IR (*v*_max_/cm^−1^): 3043 (**Ar**C–H); 1619 (C=N); 1584, 1537 (**Ar**C=C); 1306 (C–O).

### Synthesis of 2-(2,5-dimethoxyphenyl)-1*H*-benzimidazole (3c)


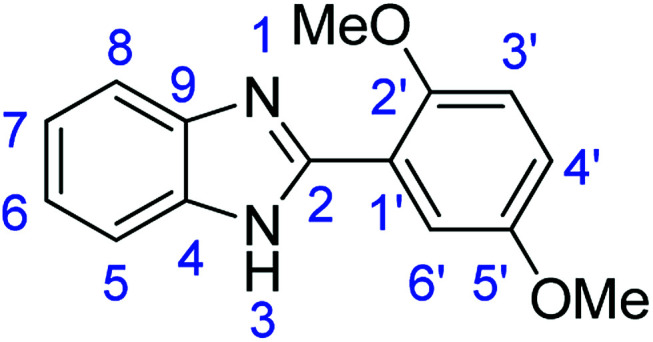


#### Method B

Stirring time = 24 h. Purification by washing with cold acetonitrile (3 × 20.0 mL) to remove any excess starting material (confirmed by TLC) to afford a brown solid (0.253 g, 10%). *R*_f_ (40% EtOAc/hexane) 0.38.

#### Method C

Stirring time = 24 h. Purification by washing with cold acetonitrile (3 × 20.0 mL) to remove any excess starting material (confirmed by TLC) to afford a brown solid (0.677 g, 25%). *R*_f_ (40% EtOAc/hexane) 0.38.

#### Method E

Novoprime Base 268 (0.105 g), stirring time = 24 h. Purification by washing with cold acetonitrile (3 × 20.0 mL) to remove any excess starting material (confirmed by TLC) to afford a white solid (1.93 g, 76%). *R*_f_ (40% EtOAc/hexane) 0.38. Mp = 224–226 °C. ([M + H]^+^ found: 255.1126 C_15_H_14_N_2_O_2_ requires [M + H]^+^, 255.1129). ^1^H NMR (500 MHz, DMSO-*d*_6_): *δ* 12.09 (1H, s, NH), 7.87 (1H, d, *J* = 2.8 Hz, H3′), 7.63 (2H, dd, *J* = 19.0, 7.2 Hz, H5, H8), 7.19 (3H, m, H6, H7, H6′), 7.05 (1H, dd, *J* = 9.0, 2.9 Hz, H4′), 3.98 (3H, s, OMe), 3.81 (3H, s, OMe). ^13^C NMR (126 MHz, DMSO-*d*_6_): *δ* 153.69 (C-2), 151.56 (C-5′), 149.21 (C-2′), 143.10 (C-9), 135.23 (C-4), 122.61 (C-7), 122.04 (C-6), 118.92 (C-1′), 117.60 (C-4′), 114.10 (C-5, C-8), 113.96 (C-2′), 112.48 (C-6′), 56.65 (OMe-5′), 56.03 (OMe-2′). IR (*v*_max_/cm^−1^): 2937 (**Ar**C–H); 1619 (C=N); 1523, 1492 (**Ar**C=C); 1301 (C–O).

### Synthesis of 2-(3,4-dimethoxyphenyl)-1*H*-benzimidazole (3d)


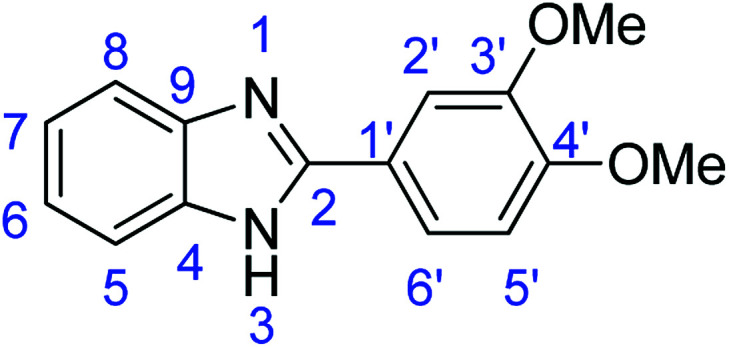


#### Method A

Stirring time = 24 h. Purification by washing with cold acetonitrile (3 × 20.0 mL) to remove any excess starting material (confirmed by TLC) to afford a brown solid (1.48 g, 59%). *R*_f_ (60% EtOAc/hexane) 0.43. Mp = 230–232 °C (lit. 41 231–232 °C). ([M + H]^+^ found: 255.1127 C_15_H_14_N_2_O_2_ requires [M + H]^+^, 255.1129). ^1^H NMR (300 MHz, chloroform-d): *δ* 8.28–7.99 (1H, m, H8), 7.74–7.43 (3H, m, H5, H2′, H6′), 7.22 (1H, d, *J* = 8.2 Hz, H7), 7.14 (1H, dd, *J* = 8.3, 3.5 Hz, H6), 7.08–6.89 (1H, m, H5′), 5.75 (1H, s, NH), 4.85–3.50 (6H, m, 2 × OMe). ^13^C NMR (126 MHz, DMSO-*d*_6_): *δ* 153.70 (C-2), 150.56 (C-3′), 149.39 (C-4′), 143.09 (C-9), 136.51 (C-4), 129.86 (C-1′), 122.97 (C-7), 122.85 (C-6), 122.51 (C-6′), 118.95 (C-8), 118.53 (C-5), 112.32 (C-2′), 111.38 (C-5′), 56.10 (2 × OMe): IR (*v*_max_/cm^−1^): 2940 (**Ar**C–H); 1606 (C=N); 1588, 1501 (**Ar**C=C); 1320 (C–O).

#### Method B

Stirring time = 24 h. Purification by washing with cold acetonitrile (3 × 20.0 mL) to remove any excess starting material (confirmed by TLC) to afford a brown solid (0.864 g, 34%). *R*_f_ (60% EtOAc/hexane) 0.42.

#### Method D

Stirring time = 24 h. Purification by washing with cold acetonitrile (3 × 20.0 mL) to remove any excess starting material (confirmed by TLC) to afford a brown solid (1.71 g, 67%). *R*_f_ (60% EtOAc/hexane) 0.42.

#### Method E

Denilite® II Base (0.085 g), stirring time = 24 h. Purification by washing with cold acetonitrile (3 × 20.0 mL) to remove any excess starting material (confirmed by TLC) to afford a yellow oil (1.45 g, 57%). *R*_f_ (60% EtOAc/hexane) 0.42.

#### Method E

Novoprime Base 268 (0.105 g), stirring time = 8 h. Purification by washing with cold acetonitrile (3 × 20.0 mL) to remove any excess starting material (confirmed by TLC) to afford a brown solid (1.97 g, 78%). *R*_f_ (60% EtOAc/hexane) 0.42.

### Synthesis of 2-phenyl-1*H*-benzimidazole (3e)


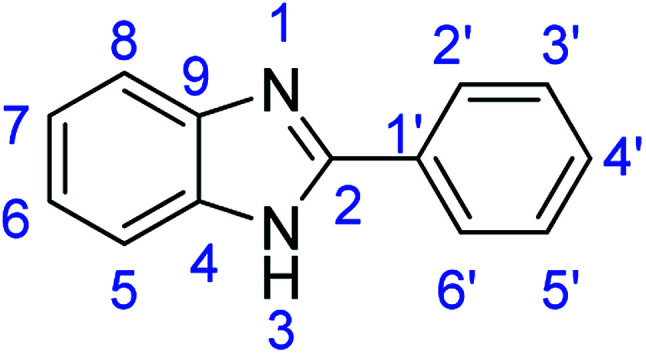


#### Method E

Novoprime Base 268 (0.105 g), stirring time = 2 h. Purification by washing with cold acetonitrile (3 × 20.0 mL) to remove any excess starting material (confirmed by TLC) to afford a brown solid (1.77 g, 92%). *R*_f_ (40% EtOAc/hexane) 0.42. Mp = 290–293 °C (lit. 40 292–293 °C). [M + H]^+^ found: 195.0916 C_13_H_10_N_2_ requires [M + H]^+^, 195.0918). ^1^H NMR (500 MHz, DMSO-*d*_6_): *δ* 12.89 (1H, s, NH), 8.19 (2H, d, *J* = 7.5 Hz, H2′, H6′), 7.68 (1H, d, *J* = 7.6 Hz, H8), 7.53 (4H, m, H5, H7, H3′, H5′), 7.21 (2H, dt, *J* = 15.6, 6.6 Hz, H6, H4′). ^13^C NMR (126 MHz, DMSO-*d*_6_): *δ* 151.69 (C-2), 144.29 (C-9, C-4), 135.47 (C-1′), 130.65 (C-4′), 130.29 (C-3′, C-5′), 129.40 (C-2′), 126.90 (C-6′), 122.99 (C-7), 122.12 (C-6), 119.34 (C-8), 111.78 (C-5). IR (*v*_max_/cm^−1^): 3047 (**Ar**C–H); 2921 (N–H); 1622 (C=N); 1559, 1542 (**Ar**C=C).

### Synthesis of 2-(2-chlorophenyl)-1*H*-benzimidazole (3f)


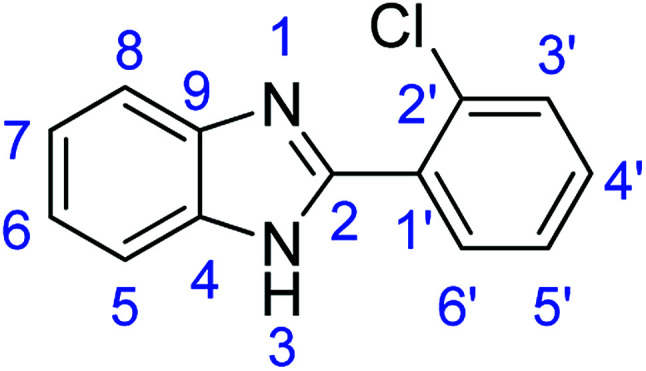


#### Method E

Novoprime Base 268 (0.105 g), stirring time = 2 h. Purification by washing with cold acetonitrile (3 × 20.0 mL) to remove any excess starting material (confirmed by TLC) to afford a brown solid (2.14 g, 94%). *R*_f_ (40% EtOAc/hexane) 0.58. Mp = 231–234 °C (lit. 40 234–235 °C). ([M + H]^+^ found: 229.0527 C_13_H_9_ClN_2_ requires [M + H]^+^, 229.0528). ^1^H NMR (500 MHz, DMSO-*d*_6_): *δ* 12.71 (1H, s, NH), 7.92 (1H, d, *J* = 7.1 Hz, H6′), 7.78–7.46 (5H, m, H5, H8, H3′, H4′, H5′), 7.25 (2H, d, *J* = 6.8 Hz, H6, H7). ^13^C NMR (126 MHz, DMSO-*d*_6_): *δ* 149.56 (C-2), 143.69 (C-1′), 135.13 (C-9), 132.54 (C-4), 132.11 (C-2′), 131.65 (C-4′), 130.81 (C-3′), 130.46 (C-6′), 127.88 (C-5′), 123.18 (C-7), 122.17 (C-6), 119.56 (C-8), 112.17 (C-5). IR (*v*_max_/cm^−1^): 3045 (**Ar**C–H); 1622 (C=N); 1569, 1539 (**Ar**C=C); 700 (C–Cl).

### Synthesis of 2-(4-chlorophenyl)-1*H*-benzimidazole (3g)


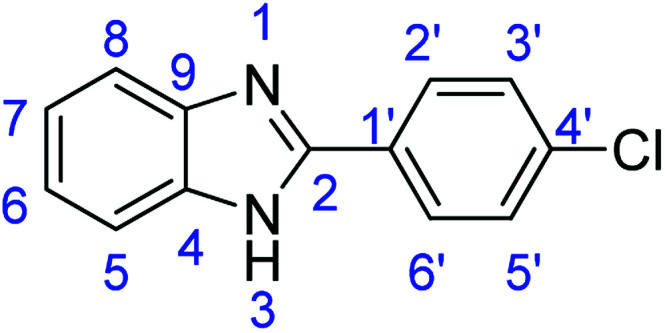


#### Method E

Novoprime Base 268 (0.105 g), stirring time = 4 h. Purification by washing with cold acetonitrile (3 × 20.0 mL) to remove any excess starting material (confirmed by TLC) to afford a brown solid (2.02 g, 88%). *R*_f_ (40% EtOAc/hexane) 0.42. Mp = 286–289 °C (lit. 42 291–294 °C). ([M + H]^+^ found: 229.0527 C_13_H_9_ClN_2_ requires [M + H]^+^, 229.0528). ^1^H NMR (500 MHz, DMSO-*d*_6_): *δ* 12.98 (1H, s, NH), 8.20 (2H, d, *J* = 8.4 Hz, H2′, H6′), 7.64 (4H, m, H5, H8, H3′, H5′), 7.22 (2H, d, *J* = 6.7 Hz, H6, H7). ^13^C NMR (126 MHz, DMSO-*d*_6_): *δ* 149.56 (C-2), 143.69 (C-9), 135.13 (C-4), 132.54 (C-4′), 132.11 (C-1′), 131.65 (C-3′), 130.81 (C-5′), 130.46 (C-2′), 127.88 (C-6′), 123.18 (C-7), 122.17 (C-6), 119.56 (C-8), 112.17 (C-5). IR (*v*_max_/cm^−1^): 3052 (**Ar**C–H); 1622 (C=N); 1588, 1540 (**Ar**C=C); 727 (C–Cl).

### Synthesis of 2-(2-nitrophenyl)-1*H*-benzimidazole (3h)


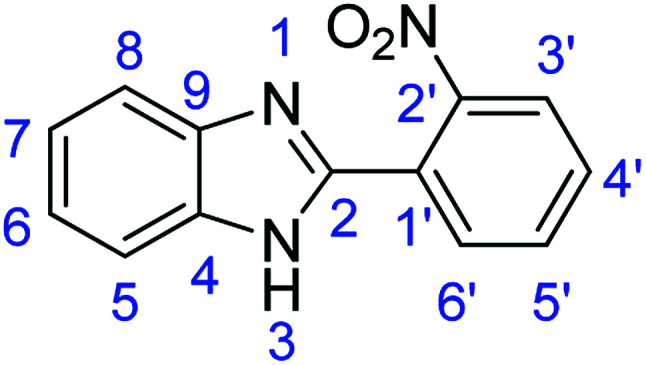


#### Method E

Novoprime Base 268 (0.105 g), stirring time = 8 h. Purification by washing with cold acetonitrile (3 × 20.0 mL) to remove any excess starting material (confirmed by TLC) to afford an orange-brown solid (1.33 g, 56%). *R*_f_ (40% EtOAc/hexane) 0.22. Mp = 191–193 °C (lit. 43 190–193 °C). ^1^H NMR (500 MHz, DMSO-*d*_6_): *δ* 12.21 (1H, s, NH), 7.21 (1H, d, *J* = 8.0 Hz, H6′), 7.15 (1H, d, *J* = 7.7 Hz, H3′), 7.04 (1H, t, *J* = 7.6 Hz, H5′), 6.94 (1H, t, *J* = 7.7 Hz, H4′), 6.79 (2H, s, H5, H8), 6.43 (2H, s, H6, H7). ^13^C NMR (126 MHz, DMSO-*d*_6_): *δ* 149.44 (C-2), 147.77 (C-2′), 133.11 (C-4, C-9), 131.39 (C-5′, C-6′), 131.36 (C-1′, C-4′), 124.76 (C-6, C-7, C-3′), 124.71 (C-5, C-8). IR (*v*_max_/cm^−1^): 3064 (**Ar**C–H); 1625 (C=N); 1573, 1524 (**Ar**C=C).

### Synthesis of 2-(pyridin-4-yl)-1*H*-benzimidazole (3i)


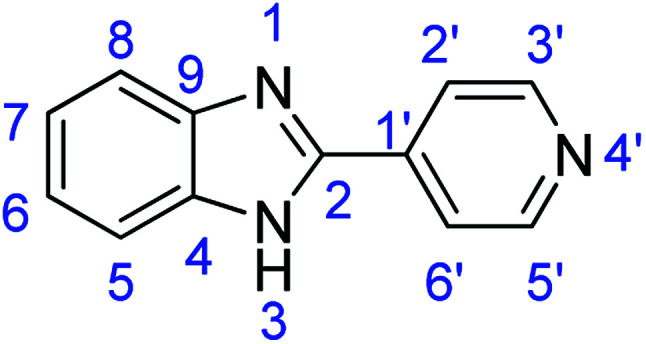


#### Method E

Novoprime Base 268 (0.105 g), stirring time = 2 h. Purification by washing with cold acetonitrile (3 × 20.0 mL) to remove any excess starting material (confirmed by TLC) to afford a brown solid (1.69 g, 60%). *R*_f_ (40% EtOAc/hexane) 0.13. Mp = 211–214 °C (lit. 42 214 °C). ([M + H]^+^ found: 196.0872 C_12_H_9_N_3_ requires [M + H]^+^, 196.0870). ^1^H NMR (500 MHz, DMSO-*d*_6_): *δ* 13.23 (1H, s, NH), 8.76 (2H, d, *J* = 4.9 Hz, H3′, H5′), 8.10 (2H, d, *J* = 4.8 Hz, H2′, H6′), 7.66 (2H, d, *J* = 68.5 Hz, H5, H8), 7.50–7.02 (2H, m, H6, H7). ^13^C NMR (126 MHz, DMSO-*d*_6_): *δ* 150.97 (C-2), 149.24 (C-3′, C-5′), 144.13 (C-1′), 137.60 (C-9), 135.49 (C-4), 124.03 (C-6, C-7), 122.73 (C-2′), 120.79 (C-6′), 119.93 (C-8), 112.27 (C-5). IR (*v*_max_/cm^−1^): 3051 (**Ar**C–H); 2877 (N–H); 1665 (C=N); 1562, 1537 (**Ar**C=C).

### Synthesis of 2-(3-nitrophenyl)-1*H*-benzimidazole (3j)


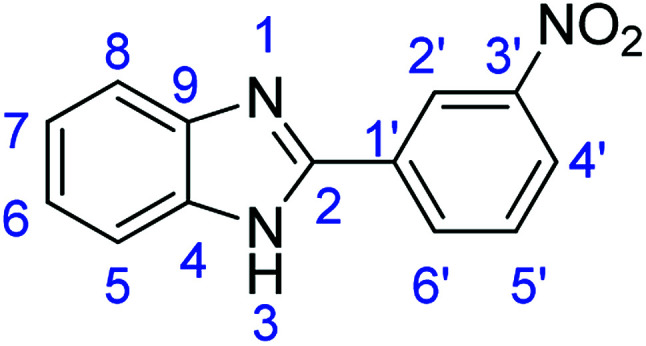


#### Method E

Novoprime Base 268 (0.105 g), stirring time = 4 h. Purification by washing with cold acetonitrile (3 × 20.0 mL) to remove any excess starting material (confirmed by TLC) to afford a brown solid (2.26 g, 95%). *R*_f_ (40% EtOAc/hexane) 0.43. Mp = 202–205 °C (lit. 43 201–203 °C). ([M + H]^+^ found: 196.0872 C_13_H_9_ClN_2_ requires [M + H]^+^, 196.0870). ^1^H NMR (500 MHz, DMSO-*d*_6_): *δ* 13.27 (1H, s, NH), 9.02 (1H, s, H2′), 8.62 (1H, d, *J* = 7.7 Hz, H6′), 8.33 (1H, d, *J* = 8.2 Hz, H4′), 7.86 (1H, t, *J* = 8.0 Hz, H5′), 7.66 (2H, d, *J* = 50.7 Hz, H5, H8), 7.26 (2H, d, *J* = 6.7 Hz, H6, H7). ^13^C NMR (126 MHz, DMSO-*d*_6_): *δ* 147.43 (C-2), 146.75 (C-3′), 130.84 (C-4, C-9), 130.12 (C-6′), 129.04 (C-1′, C-5′), 122.55 (C-4′), 119.20 (C-6, C-7, C-2′), 116.36 (C-5, C-8). IR (*v*_max_/cm^−1^): 3098 (**Ar**C–H); 1623 (C=N); 1588, 1517 (**Ar**C=C).

### Synthesis of 4-(1*H*-benzimidazol-2-yl)-*N*,*N*-dimethylaniline (3k)


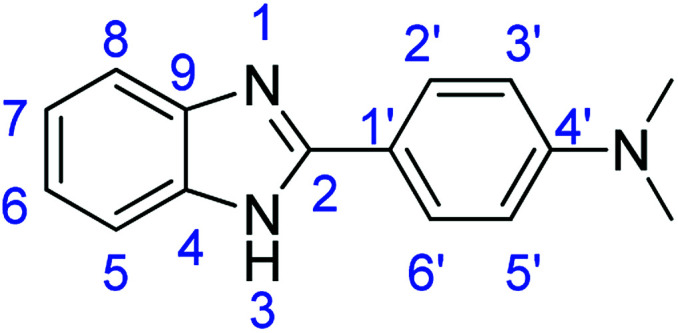


### Method E

Novoprime Base 268 (0.105 g), stirring time = 8 h. Purification by washing with cold acetonitrile (3 × 20.0 mL) to remove any excess starting material (confirmed by TLC) to afford an orange solid (1.52 g, 64%). *R*_f_ (40% EtOAc/hexane) 0.17. Mp = 201–202 °C (lit. 44 202–204 °C). ([M + H]^+^ found: 238.1339 C_15_H_15_N_3_ requires [M + H]^+^, 238.1340). ^1^H NMR (500 MHz, DMSO-*d*_6_): *δ* 8.14 (1H, s, NH), 7.99 (2H, d, *J* = 8.7 Hz, H2′, H6′), 7.48 (2H, d, *J* = 9.2 Hz, H5, H8), 7.14 (2H, d, *J* = 9.2 Hz, H6, H7), 6.83 (2H, d, *J* = 8.7 Hz, H3′, H5′), 3.00 (6H, s, 2 × N–CH_3_). ^13^C NMR (126 MHz, DMSO-*d*_6_): *δ* 163.47 (C-2), 152.72 (C-4′), 151.74 (C-9, C-4), 128.02 (C-2′, C-6′), 121.80 (C-6, C-7), 117.83 (C-5, C-8, C-1′), 112.32 (C-3′, C-5′). IR (*v*_max_/cm^−1^): 2887 (**Ar**C–H); 2773 (N–H); 1637 (C=N); 1593, 1504 (**Ar**C=C).

### Synthesis of 2-(4-nitrophenyl)-1*H*-benzimidazole (3m)


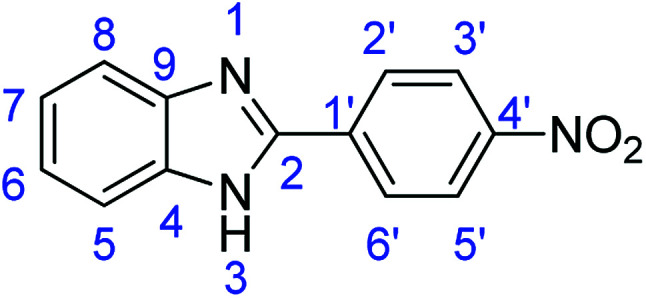


#### Method E

Novoprime Base 268 (0.105 g), stirring time = 2 h. Purification by washing with cold acetonitrile (3 × 20.0 mL) to remove any excess starting material (confirmed by TLC) to afford a brown solid (2.27 g, 95%). *R*_f_ (40% EtOAc/hexane) 0.52. Mp = 320 °C (lit. 40 >300 °C). ([M + H]^+^ found: 240.0767 C_13_H_9_N_3_O_2_ requires [M + H]^+^, 240.0769). ^1^H NMR (500 MHz, DMSO-*d*_6_): *δ* 13.28 (1H, s, NH), 8.42 (4H, s, H2′, H3′, H5′, H6′), 7.74 (1H, d, *J* = 7.6 Hz, H8), 7.59 (1H, d, *J* = 7.6 Hz, H5), 7.28 (2H, m, H6, H7). ^13^C NMR (126 MHz, DMSO-*d*_6_): *δ* 149.48 (C-2), 148.30 (C-4′), 144.32 (C-1′), 136.53 (C-9), 135.71 (C-4), 127.88 (C-2′, C-6′), 124.79 (C-3′, C-5′), 124.07 (C-7), 122.80 (C-6), 119.94 (C-8), 112.28 (C-5). IR (*v*_max_/cm^−1^): 2748 (**Ar**C–H); 1604 (C=N); 1513, 1433 (**Ar**C=C).

### Synthesis of 2-(3,4,5-trimethoxyphenyl)-1*H*-benzoimidazole (3n)


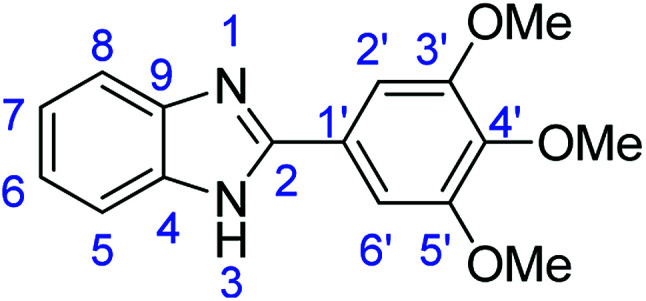


#### Method E

Novoprime Base 268 (0.105 g), stirring time = 24 h. Purification by washing with cold acetonitrile (3 × 20.0 mL) to remove any excess starting material (confirmed by TLC) to afford a white solid (2.53 g, 89%). *R*_f_ (40% EtOAc/hexane) 0.19. Mp = 258–262 °C (lit. 40 258.7–259.7 °C). ([M + H]^+^ found: 285.1230 C_16_H_16_N_2_O_3_ requires [M + H]^+^, 285.1224). ^1^H NMR (500 MHz, DMSO-*d*_6_): *δ* 12.82 (1H, s, NH), 7.66 (1H, d, *J* = 7.8 Hz, H8), 7.55–7.53 (3H, m, H5, H6, H7), 7.20 (2H, p, *J* = 7.0 Hz, H2′, H6′), 3.91 (6H, s, 2 × OMe), 3.74 (3H, s, OMe). ^13^C NMR (126 MHz, DMSO-*d*_6_): *δ* 153.69 (C-2), 151.68 (C-3′, C-5′), 144.22 (C-9), 139.38 (C-4), 135.44 (C-4′), 125.95 (C-1′), 122.89 (C-7), 122.09 (C-6), 119.17 (C-5), 111.61 (C-8), 104.30 (C-2′, C-6′), 60.62 (OMe), 56.53 (2 × OMe). IR (*v*_max_/cm^−1^): 2952 (**Ar**C–H); 1589 (C=N); 1498, 1482 (**Ar**C=C); 1311 (C–O).

### Synthesis of 2-(4-methoxyphenyl)-1*H*-benzimidazole (3o)


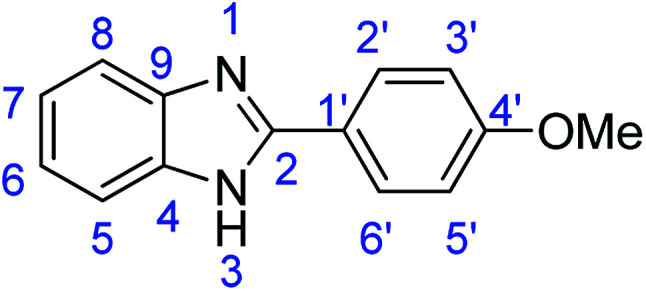


#### Method E

Novoprime Base 268 (0.105 g), stirring time = 24 h. Purification by washing with cold acetonitrile (3 × 20.0 mL) to remove any excess starting material (confirmed by TLC) to afford a light brown solid (1.53 g, 68%). *R*_f_ (40% EtOAc/hexane) 0.31. Mp = 226–228 °C (lit. 42 225–226 °C). ([M + H]^+^ found: 285.1230 C_16_H_16_N_2_O_3_ requires [M + H]^+^, 285.1024) ^1^H NMR (500 MHz, DMSO-*d*_6_): *δ* 12.73 (1H, s, NH), 8.12 (2H, d, *J* = 8.6 Hz, H2′, H6′), 7.56 (2H, d, *J* = 52.8 Hz, H5, H8), 7.20–7.14 (2H, m, H6, H7), 7.11 (2H, d, *J* = 8.6 Hz, H3′, H5′), 3.84 (3H, s, OMe). ^13^C NMR (126 MHz, DMSO-*d*_6_): *δ* 161.07 (C-2), 151.82 (C-4′), 144.37 (C-9), 135.44 (C-4), 128.48 (C-2′, C-6′), 123.19 (C-7), 122.49 (C-6), 121.98 (C-8), 121.91 (C-5), 118.96, 114.84 (C-3′, C-5′), 111.48 (C-1′), 55.80 (OMe). IR (*v*_max_/cm^−1^): 3044 (**Ar**C–H); 1621 (C=N); 1583, 1500 (**Ar**C=C); 1317 (C–O).

### Synthesis of 1-(2-methoxybenzyl)-2-(2-methoxyphenyl)-1*H*-benzimidazole (4a)


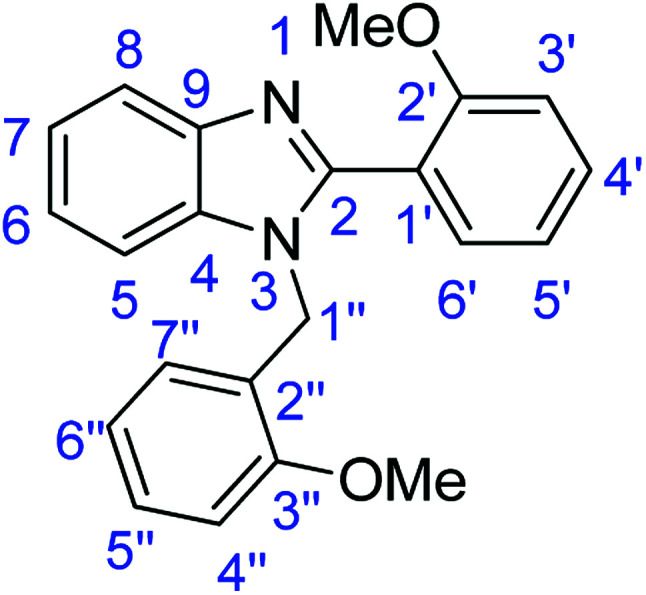


#### Method B

Stirring time = 24 h. Purification by washing with cold acetonitrile (3 × 20.0 mL) to remove any excess starting material (confirmed by TLC) to afford an orange solid (0.689 g, 20%). *R*_f_ (40% EtOAc/hexane) 0.34. Mp = 148–152 °C (lit. 44 151–153 °C). ([M + H]^+^ found: 345.1596 C_22_H_20_N_2_O_2_ requires [M + H]^+^, 334.1599). ^1^H NMR (500 MHz, DMSO-*d*_6_): *δ* 7.67 (1H, d, *J* = 7.7 Hz, H6′), 7.52 (1H, t, *J* = 7.8 Hz, H5′), 7.42 (1H, d, *J* = 7.4 Hz, H7′′), 7.36 (1H, d, *J* = 7.1 Hz, H3′), 7.27–7.12 (4H, m, H6, H7, H8, H4′), 7.07 (1H, t, *J* = 7.4 Hz, H6′′), 6.92 (1H, d, *J* = 8.2 Hz, H5), 6.75 (1H, t, *J* = 7.4 Hz, H5′′), 6.58 (1H, d, *J* = 7.4 Hz, H4′′), 5.21 (2H, s, H1′′), 3.67 (6H, s, OMe). ^13^C NMR (126 MHz, DMSO-*d*_6_): *δ* 157.53 (C-3′′), 156.83 (C-2′), 152.31 (C-2), 143.35 (C-4), 135.68 (C-9), 132.39 (C-6′), 132.02 (C-4′), 129.17 (C-7′′), 127.94 (C-5′′), 124.64 (C-2′′), 122.66 (C-7), 122.03 (C-6), 120.98 (C-5′), 120.57 (C-6′′), 120.00 (C-1′), 119.54 (C-8), 111.96 (C-5), 111.33 (C-3′), 111.16 (C-4′′), 55.75 (OMe), 55.73 (OMe), 43.25 (C-1′′). IR (*v*_max_/cm^−1^): 3063 (**Ar**C–H); 1603 (C=N); 1582, 1518 (**Ar**C=C); 1327 (C–O).

#### Method C

Stirring time = 24 h. Purification by washing with cold acetonitrile (3 × 20.0 mL) to remove any excess starting material (confirmed by TLC) to afford a brown solid (0.447 g, 13%). *R*_f_ (40% EtOAc/hexane) 0.34.

### Synthesis of 3-(1-(3-hydroxybenzyl)-1*H*-benzoimidazol-2-yl)phenol (4b)


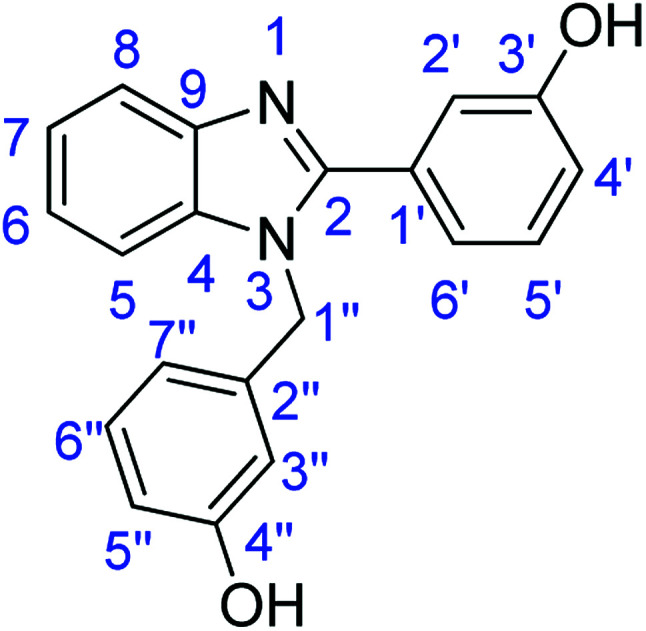


#### Method A

Stirring time = 24 h. Purification by washing with cold acetonitrile (3 × 20.0 mL) to remove any excess starting material (confirmed by TLC) to afford a brown solid (2.58 g, 82%). *R*_f_ (40% EtOAc/hexane) 0.23. Mp = 246–249 °C (lit. 45 243–245 °C). ([M + H]^+^ found: 316 C_20_H_16_N_2_O_2_ requires [M + H]^+^, 316.1286). ^1^H NMR (500 MHz, DMSO-*d*_6_): *δ* 9.77 (1H, d, *J* = 30.8 Hz, H6′), 9.43 (1H, s, H2′), 7.73 (1H, d, *J* = 7.7 Hz, H8), 7.39 (1H, d, *J* = 7.5 Hz, H5), 7.33 (1H, q, *J* = 7.7 Hz, H5′), 7.23 (3H, td, *J* = 17.7, 16.0, 8.7 Hz, H6, H7, H6′′), 7.17–7.06 (2H, m, OH), 6.95 (1H, d, *J* = 8.1 Hz, H7′′), 6.65 (1H, d, *J* = 8.0 Hz, H5′′), 6.50 (1H, d, *J* = 7.5 Hz, H4′), 6.40 (1H, s, H3′′), 5.49 (2H, s, H1′′). ^13^C NMR (126 MHz, DMSO-*d*_6_): *δ* 156.09 (C-3′), 155.91 (C-4′′), 151.69 (C-2), 140.98 (C-4), 136.71 (C-9, C-2′′), 134.25 (C-1′), 129.58 (C-5′), 128.19 (C-6′′), 120.96 (C-7), 120.52 (C-6), 117.87 (C-6′), 117.55 (C-7′′), 115.28 (C-8), 114.99 (C-5), 114.38 (C-4′), 112.82 (C-3′′), 111.07 (C-2′), 109.45 (C-5′′), 45.81 (C-1′′).

#### Method C

Stirring time = 24 h. Purification by washing with cold acetonitrile (3 × 20.0 mL) to remove any excess starting material (confirmed by TLC) to afford a brown solid (3.06 g, 97%). *R*_f_ (40% EtOAc/hexane) 0.23.

#### Method D

Stirring time = 24 h. Purification by washing with cold acetonitrile (3 × 20.0 mL) to remove any excess starting material (confirmed by TLC) to afford a brown solid (3.10 g, 98%). *R*_f_ (40% EtOAc/hexane) 0.23.

#### Method E

Novoprime Base 268 (0.105 g), stirring time = 4 h. Purification by washing with cold acetonitrile (3 × 20.0 mL) to remove any excess starting material (confirmed by TLC) to afford a brown solid (2.72 g, 86%). *R*_f_ (40% EtOAc/hexane) 0.23.

### Synthesis of 1-(2,5-dimethoxybenzyl)-2-(2,5-dimethoxyphenyl)-1*H*-benzimidazole (4c)


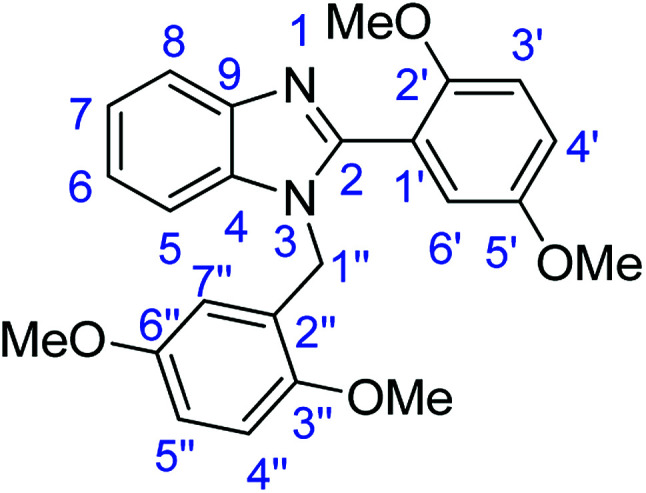


#### Method A

Stirring time = 24 h. Purification by washing with cold acetonitrile (3 × 20.0 mL) to remove any excess starting material (confirmed by TLC) to afford an oil (2.25 g, 56%). *R*_f_ (40% EtOAc/hexane) 0.36. ([M + H]^+^ found: 405.1820 C_24_H_24_N_2_O_4_ requires [M + H]^+^, 405.1810). ^1^H NMR (500 MHz, DMSO-*d*_6_): *δ* 7.73–7.57 (1H, m, H6′), 7.48–7.32 (1H, m, H8), 7.28–7.14 (2H, m, H3′, H4′), 7.12 (2H, d, *J* = 9.3 Hz, H4′′, H5′′), 7.02–6.85 (1H, m, H7), 6.85 (1H, d, *J* = 8.9 Hz, H5), 6.75 (1H, dd, *J* = 8.9, 2.7 Hz, H6), 6.15 (1H, s, H7′′), 5.19 (2H, s, H1′′), 3.74–3.45 (12H, m, 4 × OMe). ^13^C NMR (126 MHz, DMSO-*d*_6_): *δ* 153.42 (C-2), 153.32 (C-5′), 151.90 (C-6′′), 151.58 (C-3′′), 151.01 (C-2′), 143.22 (C-4), 135.63 (C-9), 125.80 (C-2′′), 122.76 (C-7), 122.10 (C-6), 120.61 (C-8), 119.56 (C-5), 117.48 (C-1′), 117.10 (C-4′), 114.80 (C-7′′), 113.23 (C-3′), 113.00 (C-6′), 112.19 (C-5′′), 111.37 (C-4′′), 56.22 (OMe-5′), 56.04 (OMe-6′′), 55.68 (OMe-3′′), 55.37 (OMe-2′), 43.26 (C-1′′).

#### Method B

Stirring time = 24 h. Purification by washing with cold acetonitrile (3 × 20.0 mL) to remove any excess starting material (confirmed by TLC) to afford an oil (3.64 g, 90%). *R*_f_ (40% EtOAc/hexane) 0.36.

#### Method C

Stirring time = 24 h. Purification by washing with cold acetonitrile (3 × 20.0 mL) to remove any excess starting material (confirmed by TLC) to afford an oil (2.82 g, 70%). *R*_f_ (40% EtOAc/hexane) 0.36.

#### Method D

Stirring time = 24 h. Purification by washing with cold acetonitrile (3 × 20.0 mL) to remove any excess starting material (confirmed by TLC) to afford an oil (3.79 g, 94%). *R*_f_ (40% EtOAc/hexane) 0.36.

### Synthesis of 1-(3,4-dimethoxybenzyl)-2-(3,4-dimethoxyphenyl)-1*H*-benzimidazole (4d)


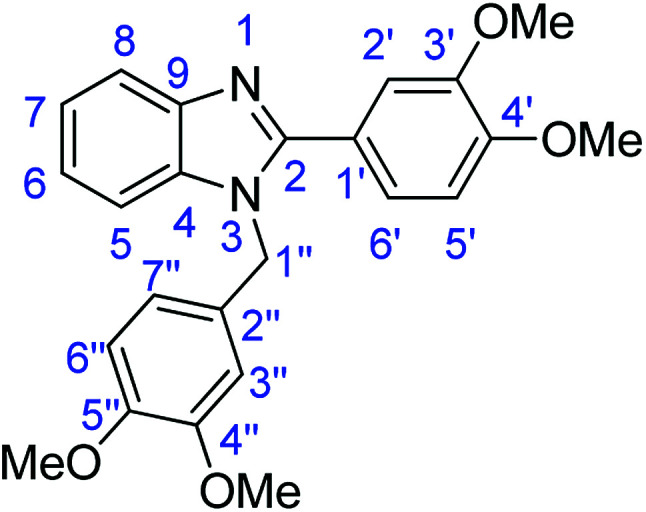


#### Method A

Stirring time = 24 h. Purification by washing with cold acetonitrile (3 × 20.0 mL) to remove any excess starting material (confirmed by TLC) to afford a yellow oil (1.45 g, 36%). *R*_f_ (60% EtOAc/hexane) 0.21. ([M + H]^+^ found: 405.1805 C_24_H_24_N_2_O_4_ requires [M + H]^+^, 405.1810). ^1^H NMR (500 MHz, DMSO-*d*_6_): *δ* 7.88 (1H, s, H2′), 7.71 (1H, d, *J* = 7.9 Hz, H6′), 7.53 (1H, d, *J* = 7.8 Hz, H8), 7.34–7.16 (3H, m, H5, H6, H7), 6.91–6.79 (2H, m, H5′, H6′′), 6.74 (1H, s, H3′′), 6.62 (1H, d, *J* = 16.3 Hz, H7′′), 5.90 (2H, m, H1′′), 3.88–3.57 (12H, m, 4 × OMe). ^13^C NMR (126 MHz, DMSO-*d*_6_): *δ* 153.71 (C-2), 150.59 (C-3′), 149.41 (C-4′), 149.36 (C-4′′), 149.18 (C-5′′), 148.60 (C-4), 143.10 (C-9), 129.87 (C-1′), 122.99 (C-7), 122.85 (C-6), 122.16 (C-2′′), 119.76 (C-6′), 119.45 (C-7′′), 118.95 (C-8), 118.56 (C-5), 112.36 (C-2′), 112.49 (C-6′), 111.37 (C-5′), 110.78 (C-3′′), 56.11 (4 × OMe), 47.74 (C-1′′). IR (*v*_max_/cm^−1^): 2970 (**Ar**C–H); 1651 (C=N); 1454 (**Ar**C=C); 1228 (C–O).

#### Method B

Stirring time = 24 h. Purification by washing with cold acetonitrile (3 × 20.0 mL) to remove any excess starting material (confirmed by TLC) to afford a yellow oil (2.66 g, 66%). *R*_f_ (60% EtOAc/hexane) 0.21.

#### Method D

Stirring time = 24 h. Purification by washing with cold acetonitrile (3 × 20.0 mL) to remove any excess starting material (confirmed by TLC) to afford a white solid (0.927 g, 23%). *R*_f_ (60% EtOAc/hexane) 0.21.

#### Method E

Denilite® II Base (0.085 g), stirring time = 24 h. Purification by washing with cold acetonitrile (3 × 20.0 mL) to remove any excess starting material (confirmed by TLC) to afford a yellow oil (1.21 g, 30%). *R*_f_ (60% EtOAc/hexane) 0.21.

### The following methods were used to synthesize benzothiazoles

#### Method F

A mixture of 2-aminothiophenol (1.63 g, 15.0 mmol) and benzaldehyde derivative (1.06 g, 10.0 mmol) in acetonitrile (10.0 mL) and acetate buffer (10.0 mL, pH 4.0) was stirred at room for 5 minutes. Suberase® (2.0 mL) was then added into the mixture and the contents were stirred for 1 h. When the reaction completes, the product precipitates from the solution and was extracted with ethyl acetate (30.0 mL) and water (3 × 20.0 mL) and concentrated on a rotary evaporator. The product was washed several times with cold acetonitrile (3 × 20.0 mL) to remove any excess starting material.

#### Method G

The same as Method A, except that methanol (2.0 mL) was used instead of acetonitrile.

#### Method H

The same as Method A, except that ethanol (4.0 mL) was used instead of acetonitrile.

#### Method I

The same as Method A, except that DMF (10.0 mL) was used instead of acetonitrile.

#### Method J

The same as Method A, except that DCM (10.0 mL) was used instead of acetonitrile.

### Synthesis of 2-phenylbenzothiazole (7a)


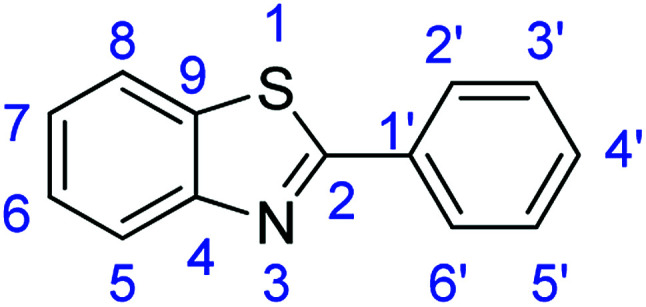


#### Method F

Stirring time = 2 h. Purification by washing with cold acetonitrile (3 × 20.0 mL) to remove any excess starting material (confirmed by TLC) to afford a bright yellow solid (1.79 g, 85%). *R*_f_ (40% EtOAc/hexane) 0.93. Mp = 96–98 °C (lit. 46 102–104 °C). ([M + H]^+^ found: 212.0528 C_13_H_9_NS requires [M + H]^+^, 212.0530). ^1^H NMR (500 MHz, chloroform-*d*): *δ* 8.15–8.04 (3H, m, H8, H2′, H6′), 7.91 (1H, d, *J* = 8.0 Hz, H5), 7.50 (4H, m, H6, H7, H3′, H5′), 7.39 (1H, t, *J* = 7.6 Hz, H4′). ^13^C NMR (126 MHz, chloroform-*d*): *δ* 154.17 (C-2), 135.08 (C-4), 133.65 (C-1′), 130.97 (C-9), 129.03 (C-2′, C-6′), 127.57 (C-5′), 126.32 (C-3′), 125.19 (C-4′), 123.26 (C-6, C-7), 121.62 (C-5, C-8). IR (*v*_max_/cm^−1^): 3075 (**Ar**C–H); 1588, 1558 (**Ar**C=C); 1644 (C=N); 727 (C–S).

#### Method G

Stirring time = 24 h. Purification by washing with cold acetonitrile (3 × 20.0 mL) to remove any excess starting material (confirmed by TLC) to afford a white solid (1.23 g, 58%). *R*_f_ (40% EtOAc/hexane) 0.93.

#### Method H

Stirring time = 24 h. Purification by washing with cold acetonitrile (3 × 20.0 mL) to remove any excess starting material (confirmed by TLC) to afford a white solid (1.16 g, 55%). *R*_f_ (40% EtOAc/hexane) 0.93.

#### Method I

Stirring time = 24 h. Purification by washing with cold acetonitrile (3 × 20.0 mL) to remove any excess starting material (confirmed by TLC) to afford a white solid (1.66 g, 78%). *R*_f_ (40% EtOAc/hexane) 0.93.

#### Method J

Stirring time = 24 h. Purification by washing with cold acetonitrile (3 × 20.0 mL) to remove any excess starting material (confirmed by TLC) to afford a white solid (1.37 g, 65%). *R*_f_ (40% EtOAc/hexane) 0.93.

### Synthesis of 4-(benzothiazol-2-yl)-N,N-dimethylaniline (7b)


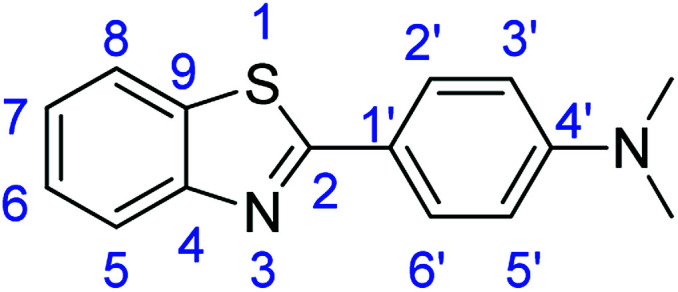


#### Method F

Stirring time = 2 h. Purification by washing with cold acetonitrile (3 × 20.0 mL) to remove any excess starting material (confirmed by TLC) to afford a cream solid (1.43 g, 56%). *R*_f_ (40% EtOAc/hexane) 0.81. Mp = 172 °C (lit. 47 170–171 °C). ([M + H]^+^ found: 255.0984 C_15_H_14_N_2_S requires [M + H]^+^, 255.0952). ^1^H NMR (500 MHz, chloroform-*d*): *δ* 8.03–7.91 (2H, m, H2′, H6′), 7.84 (1H, d, *J* = 7.9 Hz, H5), 7.44 (1H, t, *J* = 7.4 Hz, H6), 7.30 (1H, t, *J* = 7.6 Hz, H7), 6.82–6.54 (3H, m, H8, H3′, H5′), 3.01 (6H, d, *J* = 53.2 Hz, N–CH_3_). ^13^C NMR (126 MHz, chloroform-*d*): *δ* 168.79 (C-2), 154.43 (C-4′), 152.20 (C-4), 134.56 (C-9), 128.87 (C-6′), 127.71 (C-2′), 125.97 (C-6), 125.29 (C-7), 124.18 (C-1′), 122.29(C-8), 121.34 (C-5), 120.46 (C-5′), 112.29(C-3′), 40.48 (N–CH_3_), 40.18 (N–CH_3_). IR (*v*_max_/cm^−1^): 3053 (**Ar**C–H); 2904 (C–N); 2815 (N–**C–H**), 1556, 1526 (**Ar**C=C); 1604 (C=N); 720 (C–S).

### Synthesis of 2-(4-methoxyphenyl)benzothiazole (7c)


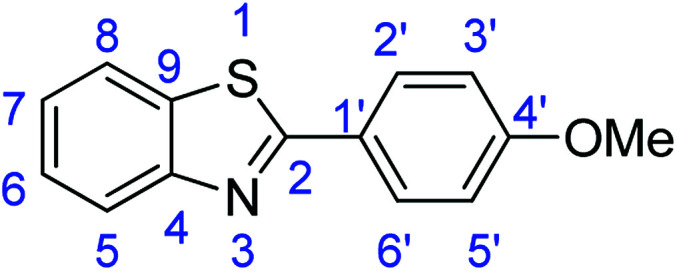


#### Method F

Stirring time = 8 h. Purification by washing with cold acetonitrile (3 × 20.0 mL) to remove any excess starting material (confirmed by TLC) to afford white solid (1.64 g, 68%). *R*_f_ (40% EtOAc/hexane) 0.89. Mp = 120–122 °C (lit. 48 120–122 °C). ([M + H]^+^ found: 242.0632 C_14_H_11_NOS requires [M + H]^+^, 242.0635). ^1^H NMR (500 MHz, chloroform-*d*): *δ* 8.04 (3H, d, *J* = 8.7 Hz, H5, H2′, H6′), 7.88 (1H, d, *J* = 8.0 Hz, H8), 7.47 (1H, t, *J* = 7.6 Hz, H6), 7.35 (1H, t, *J* = 7.6 Hz, H7), 7.00 (2H, d, *J* = 8.7 Hz, H3′, H5′), 3.88 (3H, s, OMe). ^13^C NMR (126 MHz, chloroform-*d*): *δ* 167.85 (C-2), 161.94 (C-4′), 154.24 (C-4), 134.87 (C-1′), 129.12 (C-9), 126.47 (C-6′), 126.19 (C-2′), 124.79 (C-6), 122.83 (C-7), 121.50 (C-5, C-8), 114.38 (C-3′, C-5′), 55.46 (OMe). IR (*v*_max_/cm^−1^): 3060 (**Ar**C–H); 1574, 1557 (**Ar**C=C); 1603 (C=N); 729 (C–S), 1310 (C–O).

### Synthesis of 2-(3,4-dimethoxyphenyl)benzothiazole (7d)


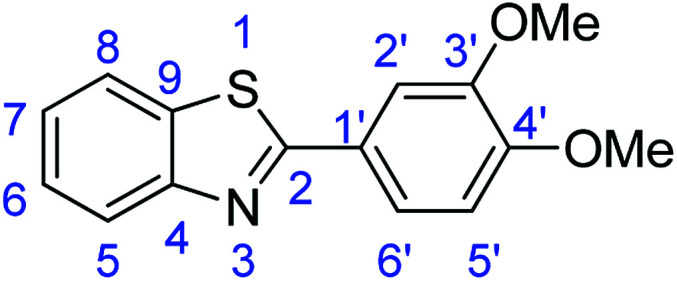


#### Method F

Stirring time = 2 h. Purification by washing with cold acetonitrile (3 × 20.0 mL) to remove any excess starting material (confirmed by TLC) to afford a cream solid (1.98 g, 73%). *R*_f_ (40% EtOAc/hexane) 0.67. Mp = 132–134 °C (lit. 48 132–134 °C). ([M + H]^+^ found: 272.0740 C_15_H_13_NO_2_S requires [M + H]^+^, 272.0741). ^1^H NMR (500 MHz, chloroform-*d*): *δ* 8.04 (1H, d, *J* = 8.2 Hz, H5), 7.88 (1H, d, *J* = 7.9 Hz, H8), 7.71 (1H, s, H2), 7.60 (1H, d, *J* = 9.5 Hz, H6′), 7.47 (1H, t, *J* = 7.7 Hz, H6), 7.36 (1H, t, *J* = 7.6 Hz, H7), 6.95 (1H, d, *J* = 8.4 Hz, H5′), 4.03 (3H, s, OMe), 3.96 (3H, s, OMe). ^13^C NMR (126 MHz, chloroform-*d*): *δ* 167.93 (C-2), 154.17 (C-4), 151.60 (C-3′), 149.37 (C-4′), 134.92 (C-9), 126.71 (C-1′), 126.23 (C-6), 124.88 (C-7), 122.85 (C-5), 121.51 (C-8), 121.16 (C-6′), 111.05 (C-2′), 109.84 (C-5′), 56.15 (OMe-3′), 56.06 (OMe-4′). IR (*v*_max_/cm^−1^): 2964 (**Ar**C–H); 1520, 1479 (**Ar**C=C); 1598 (C=N); 732 (C–S), 1312 (C–O).

### Synthesis of 2-(3,4,5-trimethoxyphenyl)benzothiazole (7e)


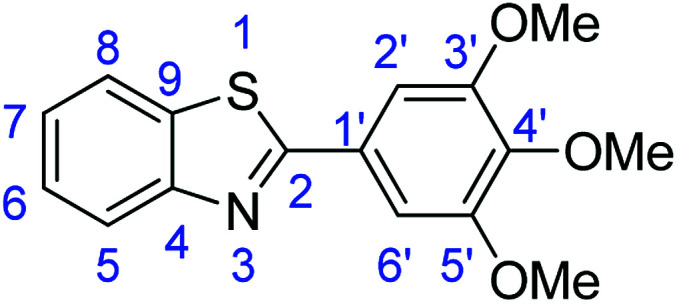


#### Method F

Stirring time = 2 h. Purification by washing with cold acetonitrile (3 × 20.0 mL) to remove any excess starting material (confirmed by TLC) to afford a yellow solid (1.96 g, 65%). *R*_f_ (40% EtOAc/hexane) 0.74. Mp = 148–151 °C (lit. 49 149.5–151.1 °C) ([M + H]^+^ found: 302.0836 C_16_H_15_NO_3_S requires [M + H]^+^, 302.0847). ^1^H NMR (500 MHz, chloroform-*d*): *δ* 8.06 (1H, d, *J* = 8.2 Hz, H5), 7.89 (1H, d, *J* = 8.0 Hz, H8), 7.49 (1H, t, *J* = 7.7 Hz, H6), 7.38 (1H, t, *J* = 7.6 Hz, H7), 7.34 (2H, s, H2′, H6′), 3.96 (9H, d, *J* = 30.7 Hz, OMe). ^13^C NMR (126 MHz, Chloroform-*d*): *δ* 167.80 (C-2), 154.10 (C-4), 153.61 (C-3′, C-5′), 140.71 (C-4′), 135.06 (C-9), 129.09 (C-1′), 126.36 (C-6), 125.13 (C-7), 123.10 (C-8), 121.56 (C-5), 104.84 (C-2′, C-6′), 61.02 (2 × OMe), 56.39 (OMe). IR (*v*_max_/cm^−1^): 2941 (**Ar**C–H); 1581, 1518 (**Ar**C=C); 1698 (C=N); 1331 (C–O); 709 (C–S).

### Synthesis of 2-(2-chlorophenyl)benzothiazole (7f)


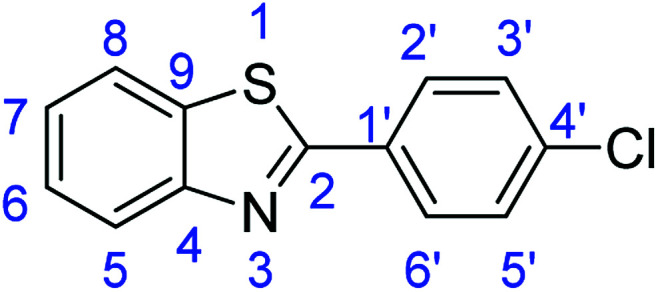


#### Method F

Stirring time = 18 h. Purification by washing with cold acetonitrile (3 × 20.0 mL) to remove any excess starting material (confirmed by TLC) to afford a cream solid (1.35 g, 55%). *R*_f_ (40% EtOAc/hexane) 0.93. Mp = 110–112 °C (lit. 50 110–112 °C). ([M + H]^+^ found: 246.0139 C_13_H_8_ClNS requires [M + H]^+^, 246.0140). ^1^H NMR (500 MHz, chloroform-*d*) *δ* 8.07 (1H, d, *J* = 8.2 Hz, H5), 8.03 (2H, d, *J* = 8.4 Hz, H2′, H6′), 7.90 (1H, d, *J* = 8.0 Hz, H8), 7.48 (3H, m, H6, H3′, H5′), 7.40 (1H, t, *J* = 7.6 Hz, H7).^13^C NMR (126 MHz, chloroform-*d*) *δ* 166.60 (C-2), 154.08 (C-5), 137.03 (C-1′), 135.06 (C-4′), 132.13 (C-9), 129.27 (C-6′), 128.71 (C-2′), 126.48 (C-3′, C-5′), 125.41 (C-6), 123.31 (C-7), 121.65 (C-5, C-8). IR (*v*_max_/cm^−1^): 3055 (**Ar**C–H); 1646 (C=N); 1588, 1569 (**Ar**C=C); 730 (C–Cl), 709 (C–S).

### Synthesis of 2-(pyridin-4-yl)benzothiazole (7g)


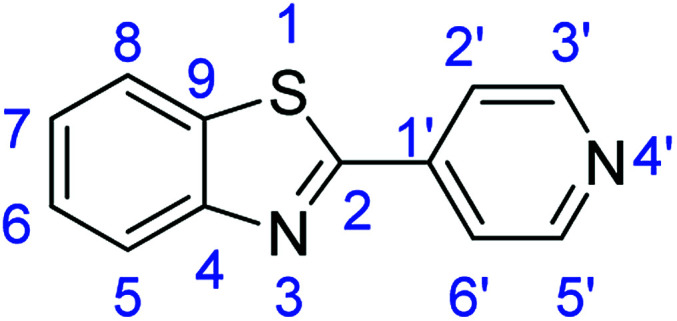


#### Method F

Stirring time = 2 h. Purification by washing with cold acetonitrile (3 × 20.0 mL) to remove any excess starting material (confirmed by TLC) to afford a bright yellow solid (1.86 g, 88%). *R*_f_ (40% EtOAc/hexane) 0.37. Mp = 126–130 °C (lit. 51 126–130 °C). ([M + H]^+^ found: 213.0481 C_12_H_8_N_2_S requires [M + H]^+^, 213.0482). ^1^H NMR (500 MHz, chloroform-*d*): *δ* 8.78 (2H, d, *J* = 5.7 Hz, H3′, H5′), 8.13 (1H, d, *J* = 8.2 Hz, H5), 7.99–7.87 (3H, m, H8, H2′, H6′), 7.55 (1H, t, *J* = 7.7 Hz, H6), 7.46 (1H, t, *J* = 7.6 Hz, H7). ^13^C NMR (126 MHz, chloroform-*d*): *δ* 165.10 (C-4), 153.99 (C-2), 150.77 (C-3′, C-5′), 140.49 (C-1′), 135.23 (C-9), 126.82 (C-6), 126.20 (C-7), 123.94 (C-8), 121.87 (C-5), 121.20 (C-2′, C-6′). IR (*v*_max_/cm^−1^): 3035 (**Ar**C–H); 1698 (C=N); 1552, 1503 (**Ar**C=C); 704 (C–S).

### Synthesis of 1,4-bis(benzothiazol-2-yl)benzene (7h)


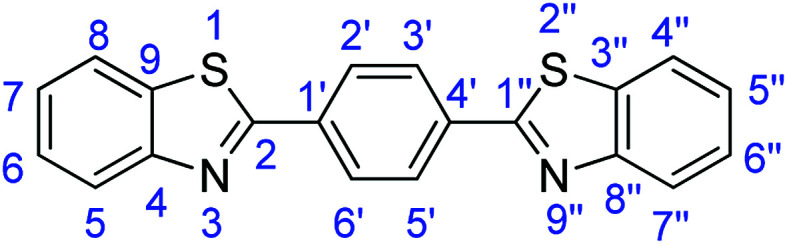


#### Method F

2-Aminothiophenol (2 equiv.), stirring time = 2 h. Purification by washing with cold acetonitrile (3 × 20.0 mL) to remove any excess starting material (confirmed by TLC) to afford a yellow solid (2.98 g, 87%). *R*_f_ (40% EtOAc/hexane) 0.81. Mp = 261–264 °C (lit. 52 263 °C). ([M + H]^+^ found: 345.0515 C_20_H_12_N_2_S_2_ requires [M + H]^+^, 345.0516). ^1^H NMR (500 MHz, chloroform-*d*): *δ* 8.23 (4H, s, H2′, H3′, H5′, H6′), 8.12 (2H, d, *J* = 8.1 Hz, H5, H7′′), 7.94 (2H, d, *J* = 8.0 Hz, H8, H4′′), 7.53 (2H, t, *J* = 7.6 Hz, H6, H6′′), 7.42 (2H, t, *J* = 7.5 Hz, H7, H5′′). ^13^C NMR (126 MHz, chloroform-*d*): *δ* 166.81(C-2, C-1′′), 154.19 (C-4, C-8′′), 135.70 (C-1′, C-4′′), 135.21 (C-9, C-3′′), 128.09 (C-2′, C-3′, C-5′, C-6′), 126.56 (C-6, C-6′′), 125.56 (C-7, C-5′′), 123.48 (C-8, C-4′′), 121.70 (C-5, C-7′′). IR (*v*_max_/cm^−1^): 3057 (**Ar**C–H); 1697 (C=N); 1558, 1522 (**Ar**C=C); 724 (C–S).

### Synthesis of 2-(2-bromo-5-methoxyphenyl)benzothiazole (7i)


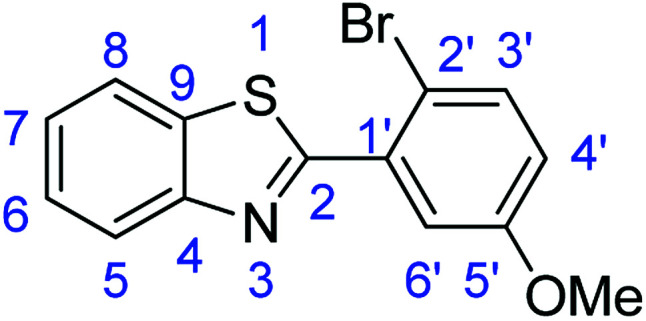


#### Method F

Stirring time = 8 h. Purification by washing with cold acetonitrile (3 × 20.0 mL) to remove any excess starting material (confirmed by TLC) to afford a yellow solid (2.42 g, 76%). *R*_f_ (40% EtOAc/hexane) 0.93. Mp = 123–125 °C. ([M + H]^+^ found: 319.9739 C_14_H_10_BrNOS requires [M + H]^+^, 319.9740). ^1^H NMR (500 MHz, chloroform-*d*): *δ* 8.68 (1H, d, *J* = 2.3 Hz, H5), 8.10 (1H, d, *J* = 8.2 Hz, H8), 7.93 (1H d, *J* = 7.9 Hz, H3′), 7.57–7.46 (2H, m, H6, H6′), 7.39 (1H, t, *J* = 7.5 Hz, H7), 6.94 (1H, d, *J* = 8.8 Hz, H4′), 4.04 (3H, s, OMe). ^13^C NMR (126 MHz, chloroform-*d*): *δ* 161.43 (C-2), 156.22 (C-5′), 152.00 (C-4), 136.20 (C-1′), 134.12 (C-9), 131.88 (C-3′), 126.11 (C-6), 124.93 (C-7), 124.05 (C-8), 122.99 (C-5), 121.24 (C-4′), 113.81 (C-6′), 113.46 (C-2′), 56.03 (OMe). IR (*v*_max_/cm^−1^): 3058 (**Ar**C–H); 1694 (C=N); 1587, 1571 (**Ar**C=C); 1315 (C–O); 751 (C–Br); 723 (C–S).

### Synthesis of 2-(3-nitrophenyl)benzothiazole (7j)


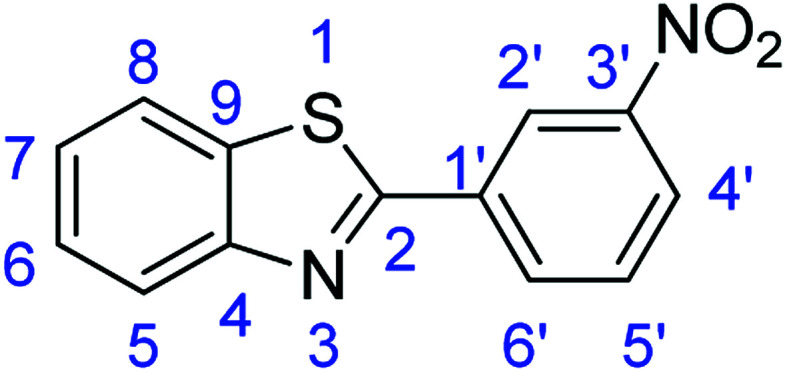


#### Method F

Stirring time = 8 h. Purification by washing with cold acetonitrile (3 × 20.0 mL) to remove any excess starting material (confirmed by TLC) to afford a yellow solid (1.23 g, 48%). *R*_f_ (40% EtOAc/hexane) 0.81. Mp = 185–187 °C (lit. 53 186.8–187.3 °C). ([M + H]^+^ found: 257.0379 C_13_H_8_N_2_O_2_S requires [M + H]^+^, 257.0380). ^1^H NMR (500 MHz, chloroform-*d*): *δ* 8.94 (1H, s, H2′), 8.43 (1H, d, *J* = 7.8 Hz, H6′), 8.34 (1H, d, *J* = 8.2 Hz, H4′), 8.12 (1H, d, *J* = 8.2 Hz, H5), 7.95 (1H, d, *J* = 8.0 Hz, H8), 7.69 (1H, t, *J* = 8.0 Hz, H5′), 7.55 (1H, t, *J* = 7.7 Hz, H6), 7.46 (1H, t, *J* = 7.6 Hz, H7). ^13^C NMR (126 MHz, chloroform-*d*): *δ* 164.89 (C-2), 153.97 (C-4), 148.79 (C-3′), 135.32 (C-6′), 135.20 (C-1′), 132.99 (C-9), 130.10 (C-5′), 126.84 (C-6), 126.03 (C-7), 125.18 (C-4′), 123.77 (C-2′), 122.35 (C-8), 121.83 (C-5). IR (*v*_max_/cm^−1^): 3085 (**Ar**C–H); 1612 (C=N); 1579, 1547 (**Ar**C=C); 1362 (N–O); 715 (C–S).

### Synthesis of 2-(2-methoxyphenyl)benzothiazole (7k)


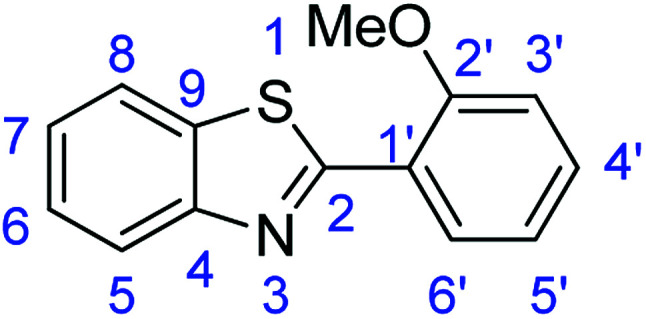


#### Method F

Stirring time = 24 h. Purification by washing with cold acetonitrile (3 × 20.0 mL) to remove any excess starting material (confirmed by TLC) to afford a brown solid (1.78 g, 74%). *R*_f_ (40% EtOAc/hexane) 0.89. Mp = 121 °C (lit. 54 120–122 °C). ([M + H]^+^ found: 242.0634 C_14_H_11_NOS requires [M + H]^+^, 242.0635). ^1^H NMR (500 MHz, chloroform-*d*): *δ* 8.54 (1H, d, *J* = 7.8 Hz, H5), 8.10 (1H, d, *J* = 8.2 Hz, H8), 7.93 (1H, d, *J* = 7.9 Hz, H6′), 7.56–7.42 (2H, m, H6, H7), 7.37 (1H, t, *J* = 7.5 Hz, H5′), 7.14 (1H, t, *J* = 7.6 Hz, H4′), 7.06 (1H, d, *J* = 8.3 Hz, H3′), 4.05 (3H, s, OMe). ^13^C NMR (126 MHz, chloroform-*d*): *δ* 153.49 (C-2), 150.51 (C-2′), 148.80 (C-4), 137.60 (C-9), 134.06 (C-4′), 133.86 (C-6′), 130.78 (C-6), 129.93 (C-7), 129.60 (C-1′), 125.66 (C-8), 123.29 (C-5), 116.28 (C-5′), 115.26 (C-3′). IR (*v*_max_/cm^−1^): 3053 (**Ar**C–H); 1604 (C=N); 1556, 1526 (**Ar**C=C); 1315 (C–O); 720 (C–S).

### Synthesis of 2-(4-nitrophenyl)benzothiazole (7l)


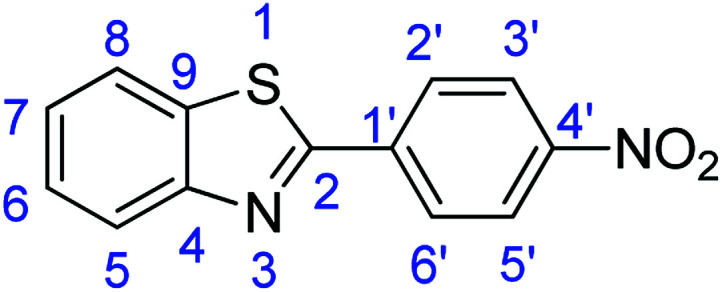


#### Method F

Stirring time = 24 h. Purification by washing with cold acetonitrile (3 × 20.0 mL) to remove any excess starting material (confirmed by TLC) to afford a yellow solid (2.15 g, 84%). *R*_f_ (40% EtOAc/hexane) 0.92. Mp = 243 °C (lit. 54 233 °C). ([M + H]^+^ found: 257.0379 C_13_H_8_N_2_O_2_S requires [M + H]^+^, 257.0380). ^1^H NMR (500 MHz, chloroform-*d*): *δ* 8.36 (2H, d, *J* = 8.7 Hz, H3′, H5′), 8.27 (2H, d, *J* = 8.7 Hz, H2′, H6′), 8.13 (1H, d, *J* = 8.2 Hz, H5), 7.96 (1H, d, *J* = 8.0 Hz, H8), 7.56 (1H, t, *J* = 7.7 Hz, H6), 7.47 (1H, t, *J* = 7.6 Hz, H7). ^13^C NMR (126 MHz, chloroform-*d*): *δ* 164.84 (C-2), 154.11 (C-4), 149.05 (C-1′), 139.19 (C-4′), 135.50 (C-9), 128.24 (C-2′, C-6′), 126.93 (C-6), 126.23 (C-7), 124.32 (C-3′), 123.95 (C-5′), 121.85 (C-5, C-8). IR (*v*_max_/cm^−1^): 3060 (**Ar**C–H); 1603 (C=N); 1574, 1557 (**Ar**C=C); 1310 (N–O); 729 (C–S).

## Conclusions

Laccases are proving themselves to be very useful oxidising catalysts that enable access to a wide range of chemicals, as was demonstrated here, synthesising pharmaceutically relevant compounds such as 2-aryl-1*H*-benzimidazoles and 2-arylbenzothiazole derivatives in good to excellent yields. This could be achieved using readily available commercial laccases, making this technique accessible to the general chemistry community.

Chemoselectivity was achieved in the synthesis of 2-aryl-1*H*-benzimidazoles by changing the solvent and laccase to Novoprime Base 268 in an acetate buffer – acetonitrile solvent. The same protocol was applied to the synthesis of 2-arylbenzothiazoles and good to excellent yields were again obtained.

The reaction proceed under very mild conditions, with low environmental impact (green), and the transformations require a simple work-up routine to obtain pure product with a simple wash with cold acetonitrile. In addition, this method eliminated the use of transition metal catalysts (*e.g.* palladium, ruthenium or copper); use of toxic oxidants such as DDQ, NiO_2_ or KI and the release of iodinated intermediates from oxidants; and the use of solvents with major environmental concerns such as DMF.

## Conflicts of interest

There are no conflicts to declare.

## Supplementary Material
